# Decreased circulating dopamine β-hydroxylase is associated with medication-related osteonecrosis of the jaw and implicates sympathetic dysregulation

**DOI:** 10.1038/s12276-026-01868-z

**Published:** 2026-10-14

**Authors:** Kyeongho Lee, Hana Cho, June Yeon Kim, Hyun Hwang, Ji-Soo Song, Soyoung Yoon, Sunbin Jung, Young-Hoon Kang, Yun Hak Kim, Jun-Young Kim, Sung-Jin Kim, Hankum Park, Jin-Woo Kim

**Affiliations:** 1 Department of Oral Histology and Developmental Biology, School of Dentistry and Dental Research Institute, Seoul National University, Seoul, 03080 Republic of Korea; 2 Department of Dental Sciences, School of Dentistry, Seoul National University, Seoul, 08826 Republic of Korea; 3 Institute for Data Innovation in Science, Seoul National University, Seoul, 08826 Republic of Korea; 4 Graduate School of Medical Sciences, Ewha Womans University, Seoul, 03760 Republic of Korea; 5 Department of Dentistry, Institute of Medical Science, Gyeongsang National University School of Medicine, Jinju, 52727 Republic of Korea; 6 Department of Anatomy, School of Medicine, Pusan National University, Yangsan, 50612 Republic of Korea; 7 Department of Oral and Maxillofacial Surgery, Yonsei University College of Dentistry, Seoul, 03722 Republic of Korea; 8 Dental Multiomics Center, Dental Research Institute, Seoul National University, Seoul, 08826 Republic of Korea; 9 Department of Oral and Maxillofacial Surgery, College of Medicine, Ewha Womans University, Seoul, 03760 Republic of Korea; 10 Ewha Dental AI & Bio Innovation Global Research Institute, Ewha Womans University, Seoul, Korea

**Keywords:** Diagnostic markers, Chronic inflammation, Proteomics, Mechanisms of disease

## Abstract

Medication-related osteonecrosis of the jaw (MRONJ) is a severe complication of antiresorptive therapy, yet no reliable circulating biomarker exists for risk stratification. Using an unbiased serum proteomics approach in a multicentre framework, we identified dopamine β-hydroxylase (DBH) as a differentially expressed protein with significantly lower levels in patients with MRONJ. This finding was validated in an independent clinical cohort and further supported in the UK Biobank population. Circulating DBH levels were positively associated with blood-pressure traits, consistent with its role as a surrogate marker of sympathetic nervous system activity, and suggesting that reduced DBH levels in MRONJ may reflect altered sympathetic regulation. In a mouse model, pharmacological sympathetic inhibition with propranolol exacerbated jaw osteonecrosis and further suppressed osteoclast activity in the presence of zoledronic acid. Osteoblast–osteoclast co-culture experiments demonstrated additive inhibitory effects on osteoclast differentiation under β-adrenergic blockade. Together, these findings support an association between reduced circulating DBH levels and MRONJ and suggest that sympathetic dysregulation may contribute to disease pathogenesis. Circulating DBH may serve as a candidate biomarker for risk assessment and disease monitoring in patients receiving antiresorptive therapy.

## Reduced Dopamine β-Hydroxylase Levels Linked to Osteonecrosis in Patients

Medication-related osteonecrosis of the jaw (MRONJ) is a serious condition linked to antiresorptive treatments, particularly in cancer patients receiving high-dose bisphosphonates. This study identifies dopamine β-hydroxylase (DBH) as a potential biomarker for MRONJ, addressing the lack of reliable circulating markers for MRONJ. Researchers used high-resolution serum proteomics to analyse samples from MRONJ patients and controls, identifying reduced DBH levels in MRONJ cases. Validation in an independent cohort and UK Biobank data confirmed these findings, showing that lower DBH levels correlate with increased MRONJ risk. The study also explored the role of sympathetic signalling in MRONJ pathogenesis, finding that propranolol, a β-adrenergic blocker, exacerbates MRONJ in a mouse model. These results suggest that DBH could serve as a biomarker for MRONJ and highlight the potential impact of sympathetic regulation on the disease.

This summary was initially drafted using artificial intelligence, then revised and fact-checked by the author.

## Introduction

Medication-related osteonecrosis of the jaw (MRONJ) is a debilitating complication primarily associated with antiresorptive agents, occasionally exacerbated by concomitant anti-angiogenic or immune-modulating medications. MRONJ is clinically defined as exposed bone, or bone that can be probed through a fistula in the maxillofacial region, persisting for more than 8 weeks in the absence of prior radiation therapy or metastatic disease to the jaws^1^. Although the incidence in patients receiving antiresorptive therapy for osteoporosis is typically below 0.2%, MRONJ occurs at substantially higher rates — often several percent or more — among cancer patients treated with high-dose intravenous bisphosphonates^2,3^. The clinical burden is considerable, including chronic bone exposure, pain and impaired mastication; in clinical practice, antiresorptive therapy is frequently paused peri-procedurally for dental interventions to mitigate MRONJ risk, which may adversely affect treatment continuity.

Despite its clinical significance, no robust or universally accepted circulating biomarkers for MRONJ have been established, posing a major obstacle to early diagnosis and risk stratification. Previous studies have proposed several candidates, including C-terminal telopeptide of type I collagen (CTx), tartrate-resistant acid phosphatase isoform 5b (TRACP 5b), dickkopf-related protein 1 (DKK1), receptor activator of nuclear factor-κB ligand (RANKL) and osteoprotegerin (OPG)^4–7^. However, none has gained widespread clinical adoption, largely because of the hypothesis-driven focus on a limited number of proteins, small and heterogeneous patient cohorts, and the lack of validation in independent populations^8^.

**Fig. 1:**
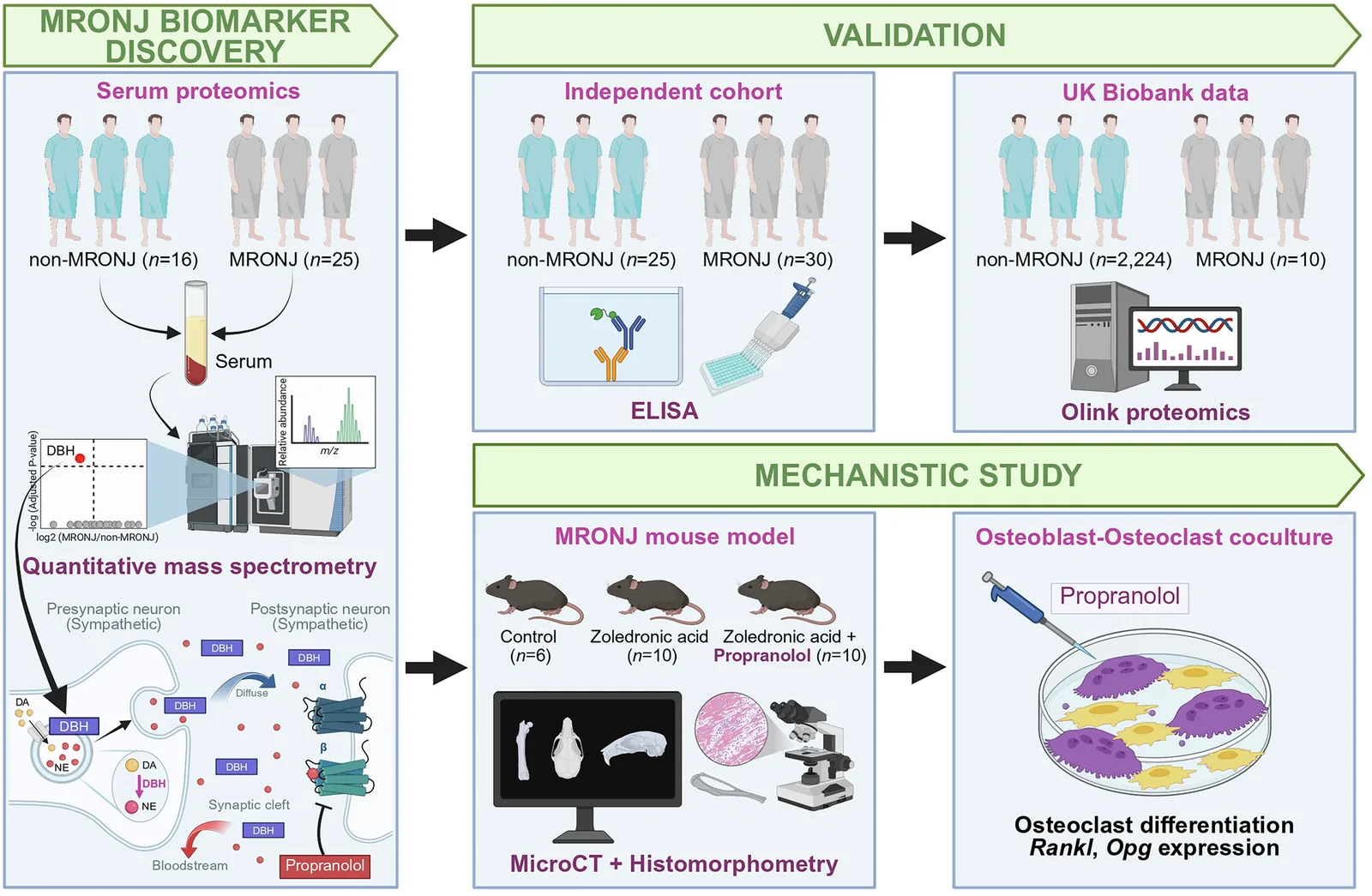
Schematic illustration of the outline of the study. This figure was created using BioRender.com. DA, dopamine; DBH, dopamine β-hydroxylase; ELISA, enzyme-linked immunosorbent assay; NE, norepinephrine; LC-MS/MS, liquid chromatography–tandem mass spectrometry; microCT, micro-computed tomography; MRONJ, medication-related osteonecrosis of the jaw; UKB, UK Biobank.

To overcome these limitations, we employed an unbiased, high-resolution serum proteomics approach to comprehensively quantify circulating proteins. The discovery findings were evaluated in an independent multicentre patient cohort and further examined using large-scale UK Biobank proteomic data, through which we identified dopamine β-hydroxylase (DBH) — a key enzyme in catecholamine biosynthesis and a surrogate marker of sympathetic activity — as a circulating protein associated with MRONJ. Its mechanistic relevance was subsequently supported by targeted in vivo and in vitro experiments, which suggested that inhibition of sympathetic signalling enhances antiresorptive effects and exacerbates MRONJ pathology. Collectively, these findings implicate DBH as a candidate circulating biomarker and support a potential link connecting sympathetic regulation with the pathogenesis of MRONJ (Fig. 1).

## Materials and methods

### Patient recruitment and blood collection

This prospective, nationwide, multicentre cohort study of patients with medication-related osteonecrosis of the jaw (MRONJ) was conducted at tertiary care centres across South Korea. Ethical approval was obtained from the Institutional Review Boards of all participating institutions, including Ewha Womanʼs University Mokdong Hospital (IRB No. EUMC 2018-04-046), Ewha Womanʼs University Seoul Hospital (IRB No. SEUMC 2023-08-042), Changwon Gyeongsang National University Hospital (IRB No. GNUCH 2022-11-11) and Yonsei University Dental Hospital (IRB No. 2-2022-0060).

Adults aged ≥20 years with a history of antiresorptive use were enrolled either in the MRONJ group, based on the 2022 American Association of Oral and Maxillofacial Surgeons (AAOMS) diagnostic criteria, or in the medication-exposed control group without clinical evidence of jaw osteonecrosis^1^. The master cohort was designed to recruit 800 participants (α = 0.05, power = 0.90), accounting for an anticipated 30% dropout rate^9^. For the present study, the discovery cohort was defined as the initial set of participants used for unbiased serum proteomic profiling to identify candidate circulating proteins associated with MRONJ. Serum samples from 41 participants (25 MRONJ patients and 16 controls without MRONJ) were analysed in this cohort. Samples were collected between January 2022 and December 2024. To verify the findings from the discovery cohort, an independent validation cohort was defined as a separate, non-overlapping set of participants recruited from the same nationwide multicentre framework. This validation cohort comprised 55 participants (30 MRONJ patients and 25 controls without MRONJ) and was analysed using identical sample collection and processing protocols.

Participants fasted overnight prior to venipuncture. Blood samples were collected between 07:00 and 10:00 h into serum separator tubes (SST; #455071, Greiner Bio-One, Austria), allowed to clot for 30 min at room temperature, and centrifuged at 1,000 × *g* for 10 min at 4 °C. Serum (1 ml) was aliquoted into 1.5-ml low-binding cryovials, snap-frozen in liquid nitrogen, and stored at −80 °C within 2 h of collection. One freeze–thaw cycle was permitted prior to proteomic analysis. All samples were processed according to a standardized operating procedure and shipped on dry ice to the central analytical laboratory. Investigators performing proteomic analyses were not blinded to group allocation. This study was registered with the Clinical Research Information Service (KCT0008077).

### Proteomic analysis

#### Serum sample preparation and protein depletion

Serum samples from MRONJ patients and controls without MRONJ were thawed on ice, vortexed and centrifuged. At the start of the workflow, equal aliquots from all serum samples were pooled to generate two global internal standard (GIS) samples for each TMT set. These GIS samples underwent the same processing as individual study samples. High-abundance serum proteins were removed using High Select Top14 Abundant Protein Depletion Mini Spin Columns (#A36369, Thermo Scientific, USA) according to the manufacturer’s instructions. Protein concentrations were determined by the Pierce Rapid Gold BCA Protein Assay Kit (#A53226, Thermo Scientific, USA) using bovine serum albumin standards.

#### Reduction, alkylation, precipitation and digestion

For each sample, 50 µg of protein was reduced with 5 mM tris (2-carboxyethyl) phosphine in 50 mM EPPS buffer (pH 8.5) at 25 °C for 15 min, followed by alkylation with 15 mM 2-chloroacetamide for 30 min. Excess alkylating agent was quenched with 15 mM dithiothreitol for 15 min. Proteins were precipitated with trichloroacetic acid and washed twice with ice-cold acetone. Pellets were resuspended in 8 M urea buffer, sonicated, and diluted to 4 M urea with EPPS buffer. Proteins were digested with Lys-C (enzyme-to-substrate ratio 1:50; #121-05063, FUJIFILM Wako,) overnight at 28 °C, further diluted to 1.2 M urea and digested with trypsin (enzyme-to-substrate ratio 1:50; #90057, Thermo Scientific, USA) for 6 h at 37 °C.

#### Sample randomization and tandem mass tag labelling

Peptide samples were randomized across three sets prior to tandem mass tag (TMT) labelling. Peptides were labelled with TMTpro 16plex reagents (#A44521, Thermo Scientific, USA) in acetonitrile for 1 h, quenched with 0.3% hydroxylamine and pooled. Each 16plex TMT set included two GIS channels to enable cross-batch normalization.

#### Peptide desalting and fractionation

Pooled, TMT-labelled peptides were desalted on Sep-Pak C18 cartridges (#WAT054955, Waters, USA). High-pH reversed-phase fractionation was performed on an Agilent Infinity 1260 HPLC system using an Aeris PEPTIDE 2.6 µm XB-C18 column (250 × 4.6 mm, #00G-4505-E0, Phenomenex, USA) with mobile phase A (10 mM ammonium bicarbonate in water/5% acetonitrile) and mobile phase B (10 mM ammonium bicarbonate in water/90% acetonitrile) at a flow rate of 0.55 ml/min. The separation was achieved over a 0–75 min gradient of mobile phase B: 3–10% (0–10 min), 10–30% (10–45 min), 30–40% (45–60 min) and 40–100% (60–75 min). Fractions were collected between 10 and 72 min into a 96-deep-well plate, concatenated (e.g. 1 + 25 + 49 + 73 … 24 + 48 + 72 + 96) to yield 24 fractions per TMT set, dried and desalted on C18 StageTips before liquid chromatography–tandem mass spectrometry (LC–MS/MS).

#### Liquid chromatography–tandem mass spectrometry analysis

Mass spectrometry was performed on an Orbitrap Exploris 480 mass spectrometer coupled to a Vanquish Neo UHPLC system (Thermo Scientific, USA). Peptides were resuspended with mobile phase A (0.1% formic acid in water) loaded onto an Acclaim PepMap trap column (150 mm × 75 µm, particle size 3 µm, bed length 20 mm; #164535, Thermo Scientific, USA) and separated on a µPAC Neo C18 analytical column (180 µm bed; 110 cm length; 2.5 × 16 µm pillar diameter; #COL-NANO110NEOB, Thermo Scientific, USA) using a gradient of mobile phase B (0.1% formic acid in acetonitrile) at a flow rate of 250 nl/min: 2.0–4.5% (0–4 min), 4.5–9.0% (4–17 min), 9.0–21.0% (17–91.5 min), 21–40% (91.5–136.5 min) and 40–90% (135.6–139.5 min).

The FAIMS Pro Interface was used with a cycle type of 1.25 s/CV with FAIMS CV of −40/−60/−80 V). MS1 full scans were acquired by Orbitrap (resolution 120,000, 375–1,500 m/z, automatic gain control target 3 × 106, maximum injection time 50 ms). Precursor fit filter (50% for a fit window 0.5 Th) was used, and precursor ions were isolated with a 0.5-Th window in the quadrupole and fragmented by normalized collision energy 36%. Dynamic exclusion (±10 ppm) was applied for 43 s. MS2 spectra were analysed with the Orbitrap (resolution 45,000, automatic gain control 7.5 × 104, maximum injection time 87 ms).

#### Database searching and quantification

RAW files were processed in Proteome Discoverer 3.1 (Thermo Scientific, USA) and searched with Sequest HT against the UniProt human reference proteome (downloaded 20 February 2024) appended with common contaminants and reversed sequences for target-decoy false discovery rate (FDR) estimation. Search parameters included a precursor ion tolerance of 10 ppm and a fragment ion tolerance of 0.02 Da. Static modifications included carbamidomethylation (+57.021 Da) at cysteine and TMTpro modification (+304.207 Da) at lysine residues. Variable modifications included oxidation at methionine (+15.995 Da), protein N-terminal acetylation (+42.011 Da) and TMTpro modification (+304.207 Da) at peptide N-termini. A maximum of two missed cleavages were allowed.

Peptide-spectrum matches were filtered to a 1% FDR using Percolator, with further filtering at the peptide and protein levels to maintain a 1% FDR. Protein quantification was based on reporter ion signal-to-noise ratios from unique and razor peptides, with normalization across TMT channels using the GIS reference channels.

Protein quantitation values were exported and analysed by R 3.6.3. Two-sided Welch’s *t* test was performed using the *t* test () function in the rstatix package version 0.7.0. Individual *P* values were adjusted for multiple testing correction, which was done using the mt.rawp2adjp() function with two-stage Benjamini–Hochberg step-up FDR-controlling procedure in the multtest package version 2.42.0.

### Multivariable logistic regression

Multivariable logistic regression was performed using the glm() function with a binomial family in R (version 4.4.3) to estimate odds ratios (ORs) and 95% confidence intervals (CIs) for MRONJ. The model included age and sex as covariates. To evaluate the risk associated with lower circulating DBH levels, DBH concentrations were standardized using the scale() function and multiplied by −1 so that effect estimates corresponded to a one-standard deviation (SD) decrease. Odds ratios and 95% CIs were calculated by exponentiating the regression coefficients and their Wald-based confidence intervals.

Sensitivity analyses were performed using the same multivariable logistic regression framework adjusted for age and sex. These analyses included the non-cancer subset, the antiresorptive-only subset after excluding participants treated with anti-angiogenic agents, the subset excluding β-blocker users and the subset excluding users of any medications that affect the sympathetic nervous system. Other medications affecting the sympathetic nervous system were defined as α-blockers, central sympatholytics, sympathomimetic agents, serotonin–norepinephrine reuptake inhibitors, tricyclic antidepressants, monoamine oxidase inhibitors or dopaminergic medications.

### Enzyme-linked immunosorbent assay

DBH enzyme-linked immunosorbent assay (ELISA) was performed according to the manufacturer’s instructions (#ab277076, Abcam, UK). Prior to ELISA, 100 μl of each serum sample — discovery cohort: MRONJ patients (*n* = 25), controls without MRONJ (*n* = 16); independent cohort: MRONJ patients (*n* = 30), controls (*n* = 25) — was transferred into clear 1.5-ml tubes, and cleared by centrifugation at 2000 × *g* for 10 min. Serum samples were diluted with the sample diluent (serum-to-diluent ratio 1:500), and heated at 70 °C for 10 min. DBH standard was prepared by serial dilution of 54,000 pg/ml DBH standard stock in the sample diluent to final concentrations ranging from 78 to 5000 pg/ml. An antibody cocktail was prepared by diluting both the capture and detector antibodies in the antibody diluent at a ratio of 1:10 for each.

For ELISA measurement, 50 μl of the diluted serum samples and DBH standards were added to a pre-coated 96-well microplate. Then, 50 μl of the antibody cocktail was added to each well, and the plate was incubated for 2 h at room temperature on a plate shaker. The plate was washed three times with 350 μl of 1X wash buffer, followed by incubation with 100 μl of TMB development solution for 10 min. The reaction was stopped by adding 100 μl of stop solution. Absorbance was measured at 450 nm (SPARK multimode microplate reader, Tecan, Switzerland).

Correlation between ELISA and mass spectrometry quantitation of DBH was assessed using Pearson’s correlation coefficient (*r*). For ELISA measurements, group differences were evaluated using the Mann–Whitney *U* test.

Receiver operating characteristic curve analysis was performed to assess the diagnostic performance of DBH concentration for distinguishing patients with MRONJ from controls. The optimal cut-off value was determined by maximizing Youden’s index (*J* = sensitivity + specificity – 1). Sensitivity and specificity were reported for the optimal cut-off, along with alternative high-sensitivity and high-specificity thresholds. Statistical analyses were conducted in GraphPad Prism 8.0.

### UK Biobank analysis

We utilized plasma proteomic data from 54,219 participants in the UK Biobank, quantified using Olink assays and expressed as normalized protein expression values on a log_2_ scale. Analyses were restricted to 44,740 participants of white British ancestry with available normalized protein expression values for DBH.

For the MRONJ analysis, 2289 participants with a diagnosis of osteoporosis or current bisphosphonate use were initially included. Participants taking medications known to influence plasma DBH levels were excluded from both case and control groups (*n* = 19). Among 2270 participants, MRONJ cases were defined as individuals diagnosed with osteonecrosis of the jaw (ICD-10 codes M87.1 or K10.2), based on previously established definitions in osteoporotic populations^10^. To avoid misclassification of controls, participants with any diagnosis of osteomyelitis or osteonecrosis (ICD-10 codes M86 or M87) were excluded from the control group (*n* = 36). Owing to the small number of MRONJ cases, Firth’s penalized likelihood logistic regression was performed using the logistf package (version 1.26.1) in R. The model was adjusted for age, sex, body mass index (BMI), bisphosphonate use and the top 10 genetic principal components. DBH levels were standardized and multiplied by −1 so that odds ratios represent the risk per one-standard deviation decrease in circulating DBH levels.

For the blood-pressure analysis cohort, 34,948 participants with available systolic blood pressure/diastolic blood pressure (SBP/DBP) measurements were included, and those with SBP ≥ 140 mmHg or DBP ≥ 90 mmHg were defined as cases of hypertension. To minimize potential confounding, 7,380 individuals taking medications known to influence plasma DBH or blood pressure were excluded from the analysis. Associations between plasma DBH levels and SBP/DBP were examined using linear, spline, quantile and logistic regression models. Partial regression plots were derived from linear models by plotting residuals of DBH and each outcome variable after adjusting for covariates. Non-linear relationships were assessed using natural cubic spline regression (df = 3) implemented with the ns() function from the splines package (version 4.3.3). Quantile regression analyses were performed across quantiles (τ = 0.05–0.95, step = 0.01) using the rq() function from the quantreg package (version 6.1), and logistic regression was used to estimate ORs for hypertension, modelling DBH either as a continuous variable or as a binary indicator for the top 30% of values. All models were adjusted for age, sex, BMI, the top 10 genetic principal components (PC1–PC10), smoking status (ever versus never), and drinking status. All analyses were conducted using R (version 4.4.3).

### Animals and in vivo experiments

Twelve-week-old male C57BL/6 J mice (20–25 g; DooYeol Biotech, Seoul, Korea) were housed under specific pathogen-free conditions at 22 °C with a 12-h light/dark cycle, with *ad libitum* access to standard rodent chow and water. All experimental procedures were approved by the Institutional Animal Care and Use Committee of Seoul National University (IACUC No. SNU-221207-2-7).

Mice were randomly assigned to three groups: (1) Control (*n* = 6), receiving intraperitoneal injections of phosphate-buffered saline and regular drinking water; (2) ZA group (*n* = 10), receiving intraperitoneal injections of zoledronic acid monohydrate (200 μg/kg; #SML0223, Sigma–Aldrich, USA) and regular drinking water; and (3) zoledronic acid + propranolol group (*n* = 10), receiving intraperitoneal injections of zoledronic acid and propranolol-containing drinking water (0.5 g/l; #P0884, Sigma–Aldrich, USA).

The MRONJ model was established using a modified protocol as previously described^11^. Briefly, mice received intraperitoneal injections of zoledronic acid three times per week until euthanasia. After 5 weeks of zoledronic acid administration, the right maxillary first, second and third molars were extracted, followed by alveoplasty to smooth sharp bony margins and standardize wound-healing conditions^12^. This procedure was performed to minimize variability in MRONJ lesion development, reduce trauma-related necrosis and more closely recapitulate clinical tooth extraction procedures in humans. Propranolol treatment was continued for 4 weeks post-extraction in the zoledronic acid + propranolol group, whereas the other groups received regular drinking water. Drinking water was replaced weekly.

At the study endpoint, mice were anaesthetized by intraperitoneal injection of Zoletil (Virbac, France), and blood was collected from the inferior vena cava. Mice were subsequently euthanized by cervical dislocation. Maxillae, femora and tibiae were harvested and fixed in 10% neutral buffered formalin (#F0160, Junsei, Japan) for 24 h at room temperature, followed by micro-computed tomography (microCT) and histological analyses.

### Histological analysis of bone necrosis and osteoclast activity

Bone necrosis was evaluated in haematoxylin and eosin (H&E)-stained sections of decalcified, formalin-fixed maxillae, following established protocols^13^. Maxillae were decalcified in ChelatorCal (BBC Biochemical, USA) at 4 °C for 3 weeks, embedded in paraffin, sectioned coronally at a thickness of 4 μm, and stained with H&E. Total bone area was measured using ImageJ software (National Institutes of Health, USA), and empty osteocyte lacunae were manually counted. The density of empty lacunae was expressed as the number per mm^2^ of total bone area (#/mm^2^). Necrotic bone was defined as regions containing ≥5 empty lacunae per mm^2^, and the proportion of necrotic bone was calculated as the necrotic area divided by the total bone area.

To assess osteoclast activity, tartrate-resistant acid phosphatase (TRAP) staining was performed on adjacent 4-μm coronal sections of decalcified maxillae using a TRAP/ALP Stain Kit (#294-67001, Wako Chemicals, Japan), according to the manufacturer’s instructions and previous reports^14^. Following deparaffinization in xylene and rehydration through graded ethanol, sections were stained and scanned using a Pannoramic MIDI II slide scanner (3DHISTECH, Hungary). TRAP-positive multinucleated osteoclasts were quantified along external bone surfaces using BioQuant OsteoImage software (BIOQUANT Image Analysis Corp., USA).

### Micro-computed tomography analysis

High-resolution microCT scanning was performed on femurs and maxillae using a Skyscan 1273 system (Bruker, Belgium) equipped with an X-ray source operating at 40–130 kV (maximum power, 40 W) and an isotropic voxel size of 12 μm. All images were reconstructed into cross-sectional slices using NRecon software (version 1.6.10.4, Bruker, Belgium). Quantitative morphometric analyses were conducted using CTAn software (version 1.22.3.1, Bruker, Belgium) to calculate trabecular bone volume fraction, trabecular number, trabecular thickness and trabecular separation. Three-dimensional volume-rendered images were generated using CTVox software (version 3.3.0 r1403, Bruker, Belgium).

For femoral analysis, trabecular bone parameters were measured in the diaphyseal region starting 5.2 mm proximal to the distal growth plate and extending 2.028 mm proximally. For maxillary analysis, a cylindrical region of interest was defined at the second molar extraction site, with a diameter of 0.51 mm and a length of 0.96 mm. Grayscale thresholds for bone segmentation were set between 62 and 255.

### Primary osteoblast culture

Primary osteoblasts were isolated from the calvariae of 1–3-day-old C57BL/6 J mice using a sequential enzymatic digestion protocol, as previously described^14^. Neonatal mice were euthanized by immersion in 70% ethanol, and five calvariae were collected per experiment. Following dissection, calvariae were washed in serum-free α-minimum essential medium (α-MEM; Gibco, #12571071, USA) and subjected to sequential enzymatic digestion in a shaking incubator (37 °C, 200 rpm) using a digestion solution containing 0.1% collagenase type VIII (Sigma–Aldrich, #C5138, USA) and 0.2% Dispase II (Gibco, #17105041, USA) prepared in serum-free α-MEM. Each digestion step was performed for 15 min; the first fraction was discarded and the subsequent three fractions were pooled.

The pooled cell suspension was filtered through a 70-μm cell strainer (Falcon, USA) and centrifuged at 2,000 rpm for 3 min. The resulting cell pellet was resuspended in complete growth medium consisting of α-MEM supplemented with 10% fetal bovine serum (Gibco, #26140079, USA) and 1% penicillin–streptomycin, and seeded at a density corresponding to one 100-mm dish per two calvariae. Cells were cultured at 37 °C in a humidified atmosphere containing 5% CO_2_, with medium changes every 2 days. When cultures reached 80–90% confluence, cells were detached by trypsinization and replated into 12-well plates (1 × 10^5^ cells per well) or 96-well plates (1 × 10^4^ cells per well) for subsequent experiments.

### Quantitative real-time reverse transcription polymerase chain reaction

Total RNA was extracted from cultured primary cells using the RNeasy Mini Kit (#74106, Qiagen, Hilden, Germany) according to the manufacturer’s instructions. RNA concentration and purity were assessed spectrophotometrically. For complementary DNA synthesis, 0.2 μg of total RNA from each sample was reverse-transcribed using the RevertAid RT Kit (#EP0441, Thermo Fisher Scientific, USA).

Quantitative real-time PCR was performed using the SensiFAST SYBR Hi-ROX Kit (#BIO-92020, Bioline, UK) on a real-time PCR detection system. Glyceraldehyde-3-phosphate dehydrogenase (*Gapdh*) was used as the internal control for normalization. Relative gene expression levels were calculated using the 2⁻ΔΔCt method. Primer sequences are provided in Supplementary Table 4.

### Osteoblast–osteoclast co-culture

Co-culture of osteoblasts and osteoclast progenitors was performed following a previously described protocol with minor modifications^15^. On day 1, primary osteoblasts were seeded into 96-well plates at a density of 1 × 10^4^ cells/well and cultured in complete medium supplemented with 30 nM 1,25-dihydroxyvitamin D₃ (Sigma-Aldrich, #D1530, USA) and 1 μM prostaglandin E_2_ (Cayman, #14010, USA).

On the following day, bone marrow-derived cells were isolated from the femurs and tibiae of 5–9-week-old C57BL/6 J mice and used as osteoclast progenitors. Briefly, femurs and tibiae were dissected under sterile conditions, and adherent soft tissues were carefully removed. After washing three times with serum-free α-MEM (Gibco, USA), the epiphyses were removed, and bone marrow cells were collected by brief centrifugation. The cell pellet was resuspended in complete growth medium and passed through a 70-μm cell strainer (#352350, Falcon, USA). After centrifugation, the pellet was resuspended in 5 ml ACK lysis buffer to remove red blood cells, diluted with 35 ml phosphate-buffered saline, and centrifuged again (1,300 rpm, 3 min). The resulting cell pellet was resuspended in complete medium, and cells were seeded onto osteoblast monolayers at a density of 1 × 10^5^ cells/well containing 30 nM 1,25-dihydroxyvitamin D_3_ and 1 μM prostaglandin E_2_ and the appropriate experimental treatments.

The culture medium was replaced with fresh medium and renewed every 2 days. After 7–10 days of co-culture, osteoclast differentiation was evaluated by TRAP staining. Images were acquired using a digital upright fluorescence microscope (#DS-Ri2/Nikon Ti, Nikon, Japan) and analysed using ImageJ software to quantify the TRAP+ osteoclast area and the number of multinucleated osteoclasts (≥3 nuclei, diameter ≥50 μm) per well.

## Results

### Clinical characteristics of discovery and validation cohorts

To identify circulating biomarkers specifically associated with the development of MRONJ among patients exposed to antiresorptive therapy, we first analysed a discovery cohort comprising 41 participants stratified by MRONJ status (patients with MRONJ, *n* = 25; controls without MRONJ, *n* = 16) using unbiased serum proteomic profiling (Table 1). Candidate proteins identified in this discovery analysis were subsequently evaluated in an independent, non-overlapping validation cohort comprising 55 participants (30 patients with MRONJ and 25 controls without MRONJ) using ELISA. Non-MRONJ controls were stringently defined as individuals who had received antiresorptive therapy and had undergone dentoalveolar interventions, including tooth extraction, during the observation period but did not develop any clinical evidence of MRONJ.

**Table 1:** Baseline clinical characteristics of patients with medication-related osteonecrosis of the jaw (MRONJ) and antiresorptive agent-exposed controls.

	Discovery cohort			Independent validation cohort		
Characteristic	Patients with MRONJ (*n* = 25)	Non-MRONJ controls (*n* = 16)	*P* value	Patients with MRONJ (*n* = 30)	Non-MRONJ controls (*n* = 25)	*P* value
**Demographics**						
Age, years	71.8 ± 9.1	75.5 ± 8.8	0.203	77.1 ± 8.9	71.0 ± 10.1	0.023
Female	24 (96)	13 (81)	0.281	25 (83)	20 (80)	1.000
**Clinical history**						
Osteoporosis	19 (76)	16 (100)	0.065	26 (87)	17 (68)	0.114
ONJ-related cancer	7 (28)	2 (13)	0.441	4 (13)	2 (8)	0.678
Hypertension	10 (40)	10 (63)	0.208	15 (50)	11 (44)	0.788
Diabetes	8 (32)	1 (6)	0.066	8 (27)	7 (28)	1.000
**Dental history**						
Tooth extraction	13 (52)	16 (100)	< 0.001	19 (63)	17 (68)	0.781
Implant	6 (24)	8 (50)	0.105	5 (17)	5 (20)	1.000
**Antiresorptive/anti-angiogenic** **medication exposure**						
Denosumab	13 (52)	7 (44)	0.751	12 (40)	7 (28)	0.404
Alendronate	4 (16)	3 (19)	1.000	5 (17)	3 (12)	0.715
Ibandronate	4 (16)	9 (56)	0.014	11 (37)	6 (24)	0.386
Zoledronic acid	6 (24)	2 (13)	0.448	3 (10)	4 (16)	0.689
Risedronate	3 (12)	4 (25)	0.401	6 (20)	0 (0)	0.027
Antiangiogenic drugs	6 (24)	0 (0)	0.065	1 (3)	0 (0)	1.000
**Antihypertensive** **medication exposure**						
β-blocker	3 (12)	1 (6)	1.000	5 (17)	1 (4)	0.204
ACE inhibitor/ARB	5 (20)	6 (38)	0.287	6 (20)	1 (4)	0.112
Calcium channel blocker	7 (28)	3 (19)	0.712	6 (20)	2 (8)	0.269
Diuretic	5 (20)	1 (6)	0.376	5 (17)	1 (4)	0.204
**Exposure to other medications affecting the sympathetic nervous system**	5 (20)	2 (13)	0.685	3 (10)	4 (16)	0.689

Values are presented as mean ± SD or number (%). Percentages were rounded to the nearest whole number. Age was compared using Welch’s *t* test; all categorical variables were compared using Fisher’s exact test (two-sided). Osteonecrosis of the jaw-related cancer was defined as prostate, breast, multiple myeloma or lung cancer treated with denosumab (Xgeva) or zoledronic acid (Zometa). ACE inhibitor/ARB indicates angiotensin-converting enzyme inhibitor or angiotensin II receptor blocker. Other medications affecting the sympathetic nervous system included α-blockers, central sympatholytics, sympathomimetic agents, serotonin–norepinephrine reuptake inhibitors, tricyclic antidepressants, monoamine oxidase inhibitors and dopaminergic medications, excluding β-blockers.

Baseline demographic and clinical characteristics were largely comparable between patients with MRONJ and controls without MRONJ, with values presented in this order throughout this section. These included age (71.8 ± 9.1 vs 75.5 ± 8.8 years; *P* = 0.203), sex (96% vs 81% female; *P* = 0.281), hypertension (40% vs 63%; *P* = 0.208) and other clinical variables. Tooth extraction within the preceding year was significantly more frequent in controls without MRONJ (100% vs 52%; *P* < 0.001), reflecting the study design. In contrast, no statistically significant differences were observed in other comorbidities or medication exposures, including diabetes (32% vs 6%; *P* = 0.066), zoledronic acid use (24% vs 13%; *P* = 0.448), denosumab use (52% vs 44%; *P* = 0.751) or anti-angiogenic therapy use (24% vs 0%; *P* = 0.065).

To validate the proteomic findings, an independent cohort of 55 participants (patients with MRONJ, *n* = 30; controls without MRONJ, *n* = 25) was analysed using ELISA. Similar to the discovery cohort, the two groups were broadly comparable in demographic and clinical characteristics. Age was slightly higher in MRONJ patients (77.1 ± 8.9 vs 71.0 ± 10.1 years; *P* = 0.023), whereas sex distribution (83% vs 80% female; *P* = 1.000), prevalence of osteoporosis (87% vs 68%; *P* = 0.114), hypertension (50% vs 44%; *P* = 0.788) and diabetes (27% vs 28%; *P* = 1.000) did not differ significantly. Patterns of antiresorptive medication exposure were largely comparable between patients with MRONJ and controls without MRONJ, including denosumab (40% vs 28%; *P* = 0.404) and zoledronic acid use (10% vs 16%; *P* = 0.689). Antihypertensive medication classes, including β-blockers, ACE inhibitors/angiotensin receptor blockers, calcium-channel blockers and diuretics, as well as other medications affecting the sympathetic nervous system, showed no significant differences between patients with MRONJ and controls without MRONJ in either cohort.

### Serum proteomic analysis identifies reduced dopamine β-hydroxylase in patients with medication-related osteonecrosis of the jaw

**Fig. 2:**
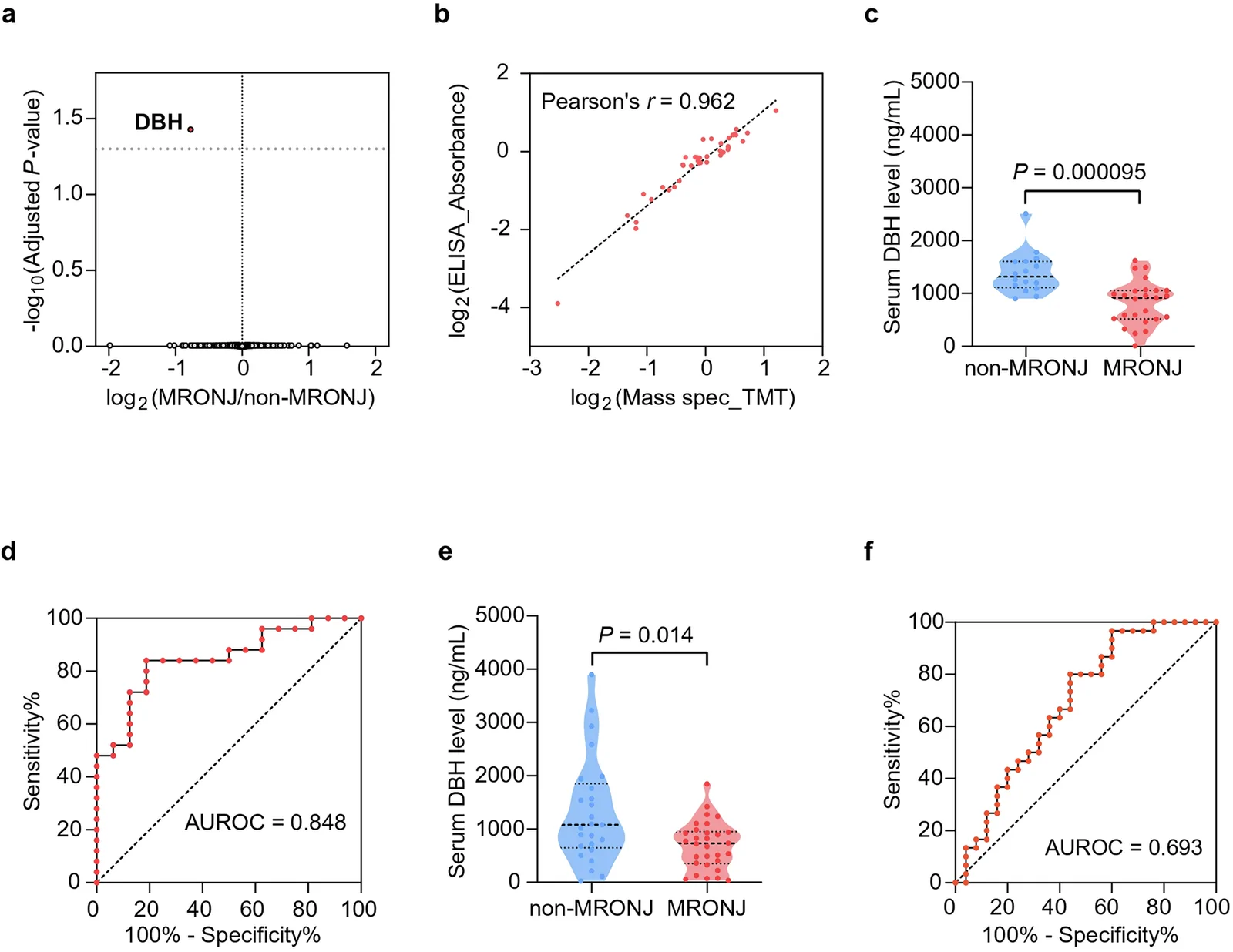
Proteomics-driven identification and multi-level validation of serum dopamine β-hydroxylase as a candidate biomarker for medication-related osteonecrosis of the jaw. **a** Volcano plot of quantified serum proteins from tandem mass tag (TMT)-based proteomics in the discovery cohort. The x-axis indicates the log_2_-fold change (patients with medication-related osteonecrosis of the jaw (MRONJ)/controls without MRONJ), and the y-axis shows the −log_10_ adjusted *P* value. The horizontal dotted line denotes the significance threshold (adjusted *P* = 0.05). Statistical significance was evaluated using a two-sided Welch’s *t* test with Benjamini–Hochberg correction. **b** Correlation between TMT-based mass spectrometry and enzyme-linked immunosorbent assay (ELISA) measurements of dopamine β-hydroxylase (DBH) in the discovery cohort. The x-axis represents log_2_-transformed TMT reporter ion intensities, and the y-axis represents log_2_-transformed ELISA absorbance values at 450 nm. Pearson’s correlation coefficient is shown. **c** Serum DBH levels measured by ELISA in the discovery cohort. Violin plots represent the distribution, with centre lines indicating the median and interquartile range. Statistical significance was assessed using a two-sided Mann–Whitney test. **d** Receiver operating characteristic curve for serum DBH levels in the discovery cohort. The dashed diagonal line indicates random classification. **e** Serum DBH levels measured by ELISA in the independent validation cohort. Violin plots represent the distribution, with centre lines indicating the median and interquartile range. Statistical significance was assessed using a two-sided Mann–Whitney test. **f** Receiver operating characteristic curve for serum DBH levels in the validation cohort. The dashed diagonal line indicates random classification. AUROC, area under the receiver operating characteristic curve.

To identify serum protein changes associated with MRONJ, we performed quantitative mass spectrometry-based proteomic profiling in 25 patients with MRONJ and 16 controls without MRONJ. Using a TMT-based workflow with abundant protein depletion, we identified and quantified 1,481 serum proteins (Supplementary Table 1). The median coefficients of variation between global internal standard channels in each TMT batch were 4.5%, 4.0% and 4.1%, indicating high technical reproducibility. Among these proteins, DBH emerged as the top differentially expressed protein, showing significantly lower abundance in patients with MRONJ than in controls without MRONJ (adjusted *P* = 0.037, Benjamini–Hochberg correction; Fig. 2a).

To validate this finding using an orthogonal antibody-based method, serum DBH levels were measured using ELISA in the same cohort. ELISA measurements strongly correlated with mass spectrometry quantitation (Pearson’s *r* = 0.962, *P* < 0.0001; Fig. 2b). Median DBH concentrations were significantly lower in patients with MRONJ (915.1 pg/ml, *n* = 25) than in controls without MRONJ (1,317 pg/ml, *n* = 16) (*P* = 9.5 × 10^−5^, Mann–Whitney test; Fig. 2c).

To evaluate whether the association between DBH levels and MRONJ status was independent of potential confounders, multivariable logistic regression analysis adjusting for age and sex was performed. Lower DBH levels were significantly associated with higher odds of MRONJ (per SD decrease, OR = 11.68, 95% CI 1.99–68.37, *P* = 0.006; Table 2). Age and sex were not significantly associated with MRONJ status.

**Table 2:** Multivariable logistic regression analysis of circulating dopamine β-hydroxylase (DBH) levels and medication-related osteonecrosis of the jaw (MRONJ) in clinical cohorts.

	Discovery cohort			Validation cohort		
Characteristic	OR	95% CI	*P* value	OR	95% CI	*P* value
DBH (per SD ↓ )	11.68	1.99–68.37	0.006	2.52	1.09–5.81	0.031
Age	0.91	0.82–1.02	0.118	1.05	0.98–1.13	0.143
Sex (female)	13.10	0.24–703.40	0.206	0.68	0.13–3.67	0.653

ORs and 95% CIs were estimated using multivariable logistic regression models adjusted for age and sex. The criterion variable was MRONJ status, with patients with MRONJ coded as the event group and controls without MRONJ as the reference group. DBH levels were standardized and modelled per 1-SD decrease. Therefore, ORs >1 indicate higher odds of MRONJ with lower circulating DBH levels. Analyses were performed separately in the discovery and independent validation cohorts.

To assess the discriminatory performance of DBH, receiver operating characteristic (ROC) analysis demonstrated good discriminatory performance, with an area under the curve (AUC) of 0.848 (95% CI 0.729–0.966, *P* = 0.0002; Fig. 2d). An optimal cut-off of 1,082 pg/ml yielded 84.0% sensitivity and 81.3% specificity (Youden’s index 0.653).

Next, DBH downregulation was independently validated by ELISA in a separate cohort of 30 patients with MRONJ and 25 controls without MRONJ. Median DBH levels were again significantly lower in patients with MRONJ (729.9 pg/ml) than in controls without MRONJ (1,082 pg/ml) (*P* = 0.014, Mann–Whitney test; Fig. 2e). Consistent with these findings, multivariable logistic regression analysis in the validation cohort demonstrated that lower DBH levels remained significantly associated with MRONJ after adjustment for age and sex (per SD decrease, OR 2.52, 95% CI 1.09–5.81, *P* = 0.031; Table 2). Age and sex were not significantly associated with MRONJ status. ROC analysis in the validation cohort demonstrated modest discrimination, with an AUC of 0.693 (Fig. 2f). The direction of effect was consistent across cohorts, although the magnitude of the association was attenuated in the validation cohort.

To further assess the robustness of the association between DBH levels and MRONJ, sensitivity analyses were performed using multivariable logistic regression models adjusted for age and sex across clinically relevant subsets. In the discovery cohort, lower DBH levels remained significantly associated with MRONJ after excluding patients with cancer (non-cancer subset: OR 7.67, 95% CI 1.45–40.58, *P* = 0.017) and after restricting the analysis to patients receiving antiresorptive agents only, excluding those treated with anti-angiogenic agents (OR 9.80, 95% CI 1.80–53.23, *P* = 0.008; Supplementary Table 2). Because medications affecting sympathetic nervous system activity could potentially confound the association between circulating DBH levels and MRONJ, we additionally performed sensitivity analyses excluding participants taking β-blockers or other medications affecting the sympathetic nervous system. In the discovery cohort, the association remained significant after excluding β-blocker users (OR 9.98, 95% CI 1.80–55.48, *P* = 0.009) and after excluding users of any medications affecting the sympathetic nervous system (OR 8.51, 95% CI 1.62–44.84, *P* = 0.012; Supplementary Table 2).

Consistent results were observed in the independent validation cohort, where lower DBH levels also remained significantly associated with MRONJ in the non-cancer subset (OR 3.29, 95% CI 1.09–9.93, *P* = 0.035) and the antiresorptive-only subset (OR 3.18, 95% CI 1.18–8.55, *P* = 0.022; Supplementary Table 2). After excluding β-blocker users, the association remained directionally consistent, although statistical significance was attenuated (OR 2.37, 95% CI 0.98–5.71, *P* = 0.055). A similar pattern was observed after excluding users of any medications affecting the sympathetic nervous system (OR 2.19, 95% CI 0.87–5.51, *P* = 0.094; Supplementary Table 2), probably reflecting the reduced sample size. These findings were further supported by subgroup comparisons of serum DBH levels, which showed consistently lower DBH concentrations in MRONJ patients across sensitivity subsets in both cohorts (Supplementary Fig. 1). Together, these results support an association between reduced circulating DBH levels and MRONJ across cohorts and clinically relevant subsets.

### Population-based plasma proteomic analysis replicates reduced dopamine β-hydroxylase levels in patients with medication-related osteonecrosis of the jaw

To further validate the reduction of serum DBH levels observed in patients with MRONJ, we analysed data from the UK Biobank, a large UK-based population cohort of approximately 500,000 individuals. Among 54,219 participants with available plasma proteomic data, analyses were restricted to individuals of white British ancestry to reduce population stratification, leaving 44,740 participants with plasma DBH protein measurements. To minimize case misclassification, we further limited the analysis to participants diagnosed with osteoporosis or receiving bisphosphonate therapy. Individuals taking medications known to influence plasma DBH levels were excluded, yielding 2,270 eligible participants. To avoid misclassification of controls, participants with any diagnosis of osteomyelitis or osteonecrosis (ICD-10 codes M86 or M87) were excluded from the control group only, resulting in a final analytical sample of 10 MRONJ cases and 2,224 controls (Supplementary Fig. 2). The prevalence of MRONJ in this UK Biobank subset was 0.45%, consistent with reported MRONJ prevalence in populations exposed to antiresorptive therapy^1^.

Clinical characteristics of the UK Biobank cohort are summarized in Supplementary Table 3. Age (59.9 ± 9.2 vs 61.7 ± 6.1 years; *P* = 0.554) and sex distribution (70% vs 81.2% female; *P* = 0.411) of the two groups were comparable. The prevalence of comorbidities, including hypertension (50% vs 60.1%; *P* = 0.533) and diabetes (10% vs 4.5%; *P* = 0.374), did not differ significantly. Osteoporosis was present in most participants (90% vs 83.2%; *P* = 1.000). Patterns of bisphosphonate exposure were similar in the two groups, with alendronate being the most frequently prescribed agent (20% vs 24.2%; *P* = 1.000). Overall bisphosphonate use did not differ significantly between patients with MRONJ and controls without MRONJ (30% vs 32.8%; *P* = 1.000).

In multivariable logistic regression analysis using Firth’s penalized likelihood approach and adjusted for age, sex, BMI, bisphosphonate use and the top 10 genetic principal components, plasma DBH levels were significantly associated with MRONJ occurrence (OR per 1-SD decrease 1.57, 95% CI 1.02–2.20, *P* = 0.042; Table 3). Age (OR 0.95, 95% CI 0.88–1.05, *P* = 0.289), BMI (OR 1.06, 95% CI 0.94–1.16, *P* = 0.326) and sex (OR 1.87, 95% CI 0.45–6.32, *P* = 0.355) were not significantly associated with MRONJ. These population-based findings indicate that lower circulating DBH levels were associated with higher odds of MRONJ, with a direction of effect consistent with that observed in both the discovery and independent validation cohorts.

**Table 3:** Logistic regression analysis of circulating dopamine β-hydroxylase (DBH) levels and medication-related osteonecrosis of the jaw (MRONJ) occurrence in UK Biobank data.

Variable	MRONJ patients (*n* = 10)	Controls without MRONJ (*n* = 2,224)	OR*	95% CI	*P* value
DBH (per SD↓)	−0.96 (1.97)	−0.18 (1.12)	1.57	1.02–2.20	0.042
Age	59.90 (9.24)	61.70 (6.09)	0.95	0.88–1.05	0.289
BMI	28.18 (6.76)	26.44 (5.05)	1.06	0.94–1.16	0.326
Sex (female)	7 (70.0%)	1,806 (81.2%)	1.87	0.45–6.32	0.355
Bisphosphonate use	3 (30%)	730 (32.8%)	0.98	0.24–3.25	0.968

Values are presented as mean ± SD or number (%). *ORs and 95% CIs were estimated using multivariable Firth’s penalized likelihood logistic regression to account for the small number of MRONJ cases, adjusting for age, sex, BMI, bisphosphonate use, and the top 10 genetic principal components. Continuous variables (age, BMI) were modelled per 1-unit increase, whereas DBH was modelled per 1-SD decrease. *P* values for group comparisons were calculated using Welch’s *t* test for continuous variables and Fisher’s exact test for categorical variables.

### Circulating dopamine β-hydroxylase level serves as a surrogate biomarker of sympathetic nervous system activity

DBH, an enzyme localized in sympathetic neurons and adrenal chromaffin cells, catalyses the conversion of dopamine to norepinephrine and thus regulates catecholaminergic neurotransmission and systemic sympathetic activity. Because it is co-released with norepinephrine during sympathetic activation, circulating DBH has been proposed as a surrogate marker of sympathetic tone^16,17^. However, findings from small-scale studies have been inconsistent owing to great interindividual variation and limited responsiveness of serum DBH to sympathetic stimuli^18,19^. To overcome these limitations, we analysed the association between plasma DBH levels and blood pressure in the UK Biobank to evaluate whether circulating DBH is associated with sympathetic tone at the population level.

**Fig. 3:**
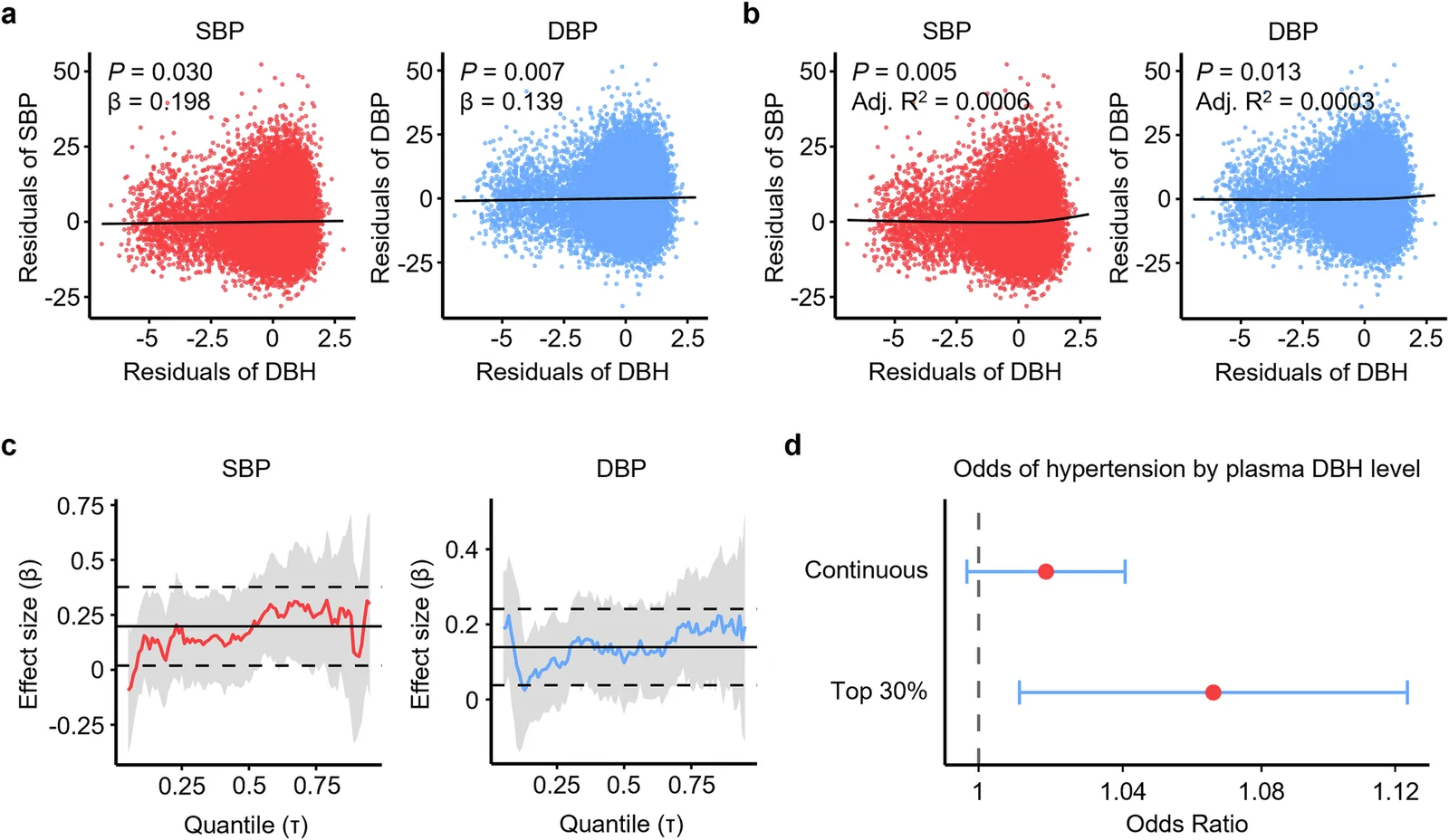
Association of plasma dopamine β-hydroxylase levels with blood-pressure traits and hypertension risk in the UK Biobank. **a**, **b** Partial regression plots showing the association between plasma dopamine β-hydroxylase (DBH) levels and systolic (SBP) or diastolic blood pressure (DBP). Both axes display residuals from linear models. The black line represents a linear fit in (**a**) and a natural cubic spline fit (df = 3) in **b**. **c** Estimated regression coefficients (β) from quantile regression analyses across quantiles τ = 0.05–0.95 (step = 0.01). The grey ribbon indicates the 95% confidence interval (CI). The horizontal black solid line represents the β estimate from (**a**) and dashed lines denote its 95% CI. **d** ORs for hypertension by plasma DBH level from logistic regression. “Continuous” models DBH as a continuous variable, whereas “Top 30%” compares participants in the top 30% of DBH levels with all others. Dots represent ORs; whiskers show 95% CIs. All models were adjusted for age, sex, BMI, the top 10 genetic principal components, smoking status (ever versus never) and drinking status.

In the UK Biobank cohort, plasma DBH levels were positively associated with both SBP and DBP after adjusting for age, sex, BMI, smoking status, drinking status and the genetic principal components (Fig. 3a). To examine potential non-linearity, spline regression analysis was performed and revealed a modest but statistically significant deviation from linearity, indicating that the association between DBH and blood pressure strengthened slightly at higher DBH levels (Fig. 3b). Quantile regression further showed that the effect size increased towards the upper quantiles of the blood-pressure distribution, suggesting that sympathetic influence associated with DBH may be more pronounced among individuals with higher blood pressure (Fig. 3c). In logistic regression, continuous DBH levels showed a marginal association with hypertension risk (*P* = 0.095), whereas participants in the top 30% of DBH levels exhibited significantly higher odds of hypertension (*P* = 0.018) (Fig. 3d). These findings indicate that circulating DBH is associated with blood pressure in a manner consistent with its proposed role as a surrogate marker of sympathetic tone.

### Sympathetic inhibition by propranolol exacerbates medication-related osteonecrosis of the jaw in a mouse model

**Fig. 4:**
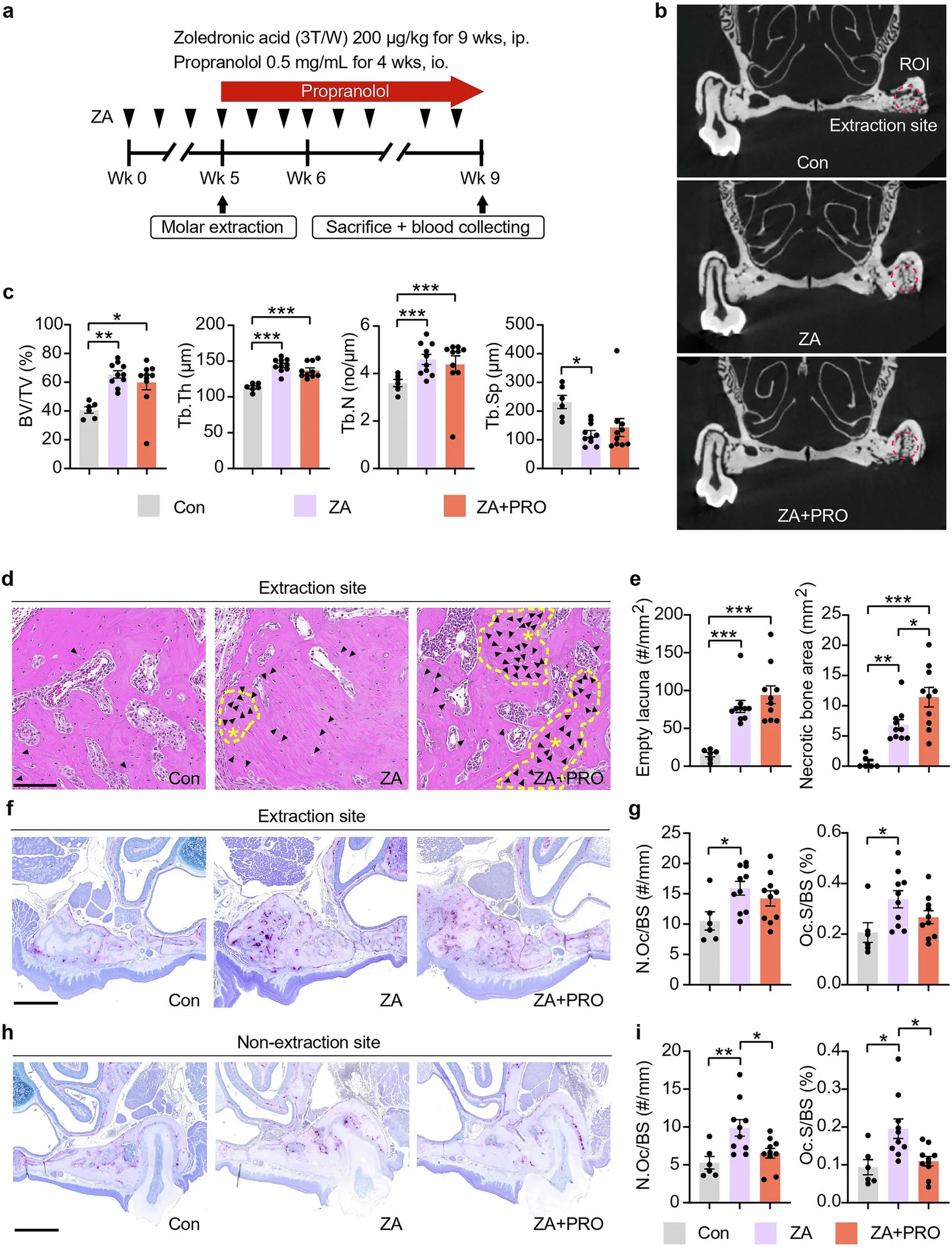
Propranolol treatment exacerbates medication-related osteonecrosis of the jaw lesion in zoledronic acid-induced medication-related osteonecrosis of the jaw mouse model. **a** Experimental timeline. Mice received zoledronic acid (ZA; 200 µg/kg, intraperitoneally, three times per week for 9 weeks) and/or propranolol (PRO; 0.5 mg/ml in drinking water for 4 weeks starting after maxillary molar extraction). Maxillary molar extraction was performed at week 5, followed by sacrifice and blood collection at week 9. **b** Representative micro-computed tomography (microCT) images of extraction and non-extraction sites in control (Con), ZA, and ZA + PRO groups. The region of interest (ROI; dashed red circle) was defined at the second molar extraction site (0.51 mm in diameter and 0.96 mm in length) to evaluate new bone formation. **c** Quantitative microCT analysis of trabecular bone parameters in the extraction socket, including bone volume fraction (BV/TV), trabecular thickness (Tb.Th), trabecular number (Tb.N), and trabecular separation (Tb.Sp). **d** Representative haematoxylin and eosin (H&E)-stained sections of extraction sites in the Con, ZA and ZA + PRO groups. Black arrowheads indicate empty lacunae, and yellow dashed outlines denote necrotic bone areas. **e** Quantitative histological assessment of medication-related osteonecrosis of the jaw-like lesions, including the number of empty lacunae (per mm^2^) and necrotic bone area (mm^2^). **f** Representative tartrate-resistant acid phosphatase (TRAP)-stained sections of extraction sites in the Con, ZA and ZA + PRO groups. **g** Quantification of TRAP-positive osteoclasts in extraction sites, expressed as osteoclast number per bone surface (N.Oc/BS) and osteoclast surface per bone surface (Oc.S/BS). **h** Representative TRAP-stained sections of non-extraction sites in the Con, ZA and ZA + PRO groups. **i** Quantification of TRAP-positive osteoclasts in non-extraction sites (N.Oc/BS and Oc.S/BS). Data are presented as mean ± SEM. *P < 0.05, **P < 0.01, ***P < 0.001.

The observed reduction in circulating DBH levels, together with its significant correlation with blood pressure, suggests altered sympathetic regulation in patients with MRONJ. However, this association alone does not establish whether impaired sympathetic signalling contributes functionally to MRONJ pathogenesis. To address this question, we pharmacologically suppressed β-adrenergic signalling using propranolol, a non-selective β-adrenergic receptor antagonist, in an established mouse model of MRONJ induced by repeated administration of zoledronic acid combined with maxillary molar extraction^11^ (Fig. 4a).

To evaluate both systemic bone homeostasis and local bone structural changes, we first performed microCT analyses of the maxillary alveolar sockets and femora. This analysis revealed significant increases in bone volume at both the extraction sockets and the femora in zoledronic acid-treated mice (Fig. 4b, c and Supplementary Fig. 3a,b), consistent with previous studies showing that zoledronic acid alone can increase bone volume at extraction sites and in long bones^20,21^. However, co-administration of propranolol with zoledronic acid did not result in further changes in bone structure, probably because the high zoledronic acid dose used in this study (200 μg/kg, three times per week) exerted such a strong antiresorptive effect that it masked any potential structural contribution from propranolol (Fig. 4b, c and Supplementary Fig. 3a, b).

Next, we investigated osteonecrotic changes in histological preparations. Histological examination revealed a marked increase in necrotic bone area — the hallmark feature of MRONJ — in mice treated with zoledronic acid compared with controls, confirming that our model recapitulates this key pathological feature of MRONJ (Fig. 4d, e). Notably, co-administration of propranolol with zoledronic acid resulted in a significantly greater necrotic area than zoledronic acid treatment alone, indicating that propranolol-induced sympathetic inhibition further exacerbates MRONJ-associated bone necrosis (Fig. 4d, e).

Given that the antiresorptive effect of zoledronic acid is a well-recognized risk factor for MRONJ, we next examined whether propranolol exerts an additional antiresorptive effect beyond zoledronic acid treatment alone using TRAP staining. Despite the potent antiresorptive action of zoledronic acid, its administration resulted in a significant increase in both the number and surface area of TRAP-positive osteoclasts at the extraction and non-extraction sites, as well as in the tibia, compared with controls (Fig. 4f–i and Supplementary Fig. 3c, d). This paradoxical accumulation of osteoclasts is consistent with previous reports showing that repeated bisphosphonate administration leads to giant, dysfunctional osteoclasts in both humans and mice, particularly at extraction sites^22–28^. Correspondingly, the serum bone resorption marker CTX-I was decreased in the zoledronic acid group, confirming that osteoclastic activity was effectively suppressed, despite the increased osteoclast numbers (Supplementary Fig. 3e).

We next investigated whether propranolol exerts additional antiresorptive effects beyond zoledronic acid treatment. Co-treatment with propranolol significantly reduced the number of giant, dysfunctional osteoclasts induced by zoledronic acid at the non-extraction site, probably by preventing osteoclast formation through an independent mechanism (Fig. 4h, i). However, propranolol did not attenuate osteoclast accumulation at the extraction site, where zoledronic acid-induced necrosis and inflammation predominated (Fig. 4f, g). Consistent with this localized effect, propranolol also failed to alter osteoclast burden in the tibia or systemic bone resorption, as measured by serum CTX-I, indicating that alveolar bone — with its higher remodelling dynamics — may be uniquely susceptible to modulation by antiresorptive agents^29^.

Collectively, these findings support the conclusion that propranolol-mediated suppression of sympathetic tone exacerbates MRONJ-associated osteonecrosis, potentially through mechanisms that include enhanced antiresorptive effects.

### Propranolol and zoledronic acid exhibit additive inhibitory effects on osteoclast differentiation

To elucidate the effects of sympathetic inhibition on osteoblast-mediated osteoclastogenic signalling, primary calvarial osteoblasts were treated with propranolol, isoproterenol and zoledronic acid, either alone or in combination. Isoproterenol was used to mimic physiological sympathetic stimulation, thereby establishing a β-adrenergic signalling context for evaluating the inhibitory effect of propranolol.

**Fig. 5:**
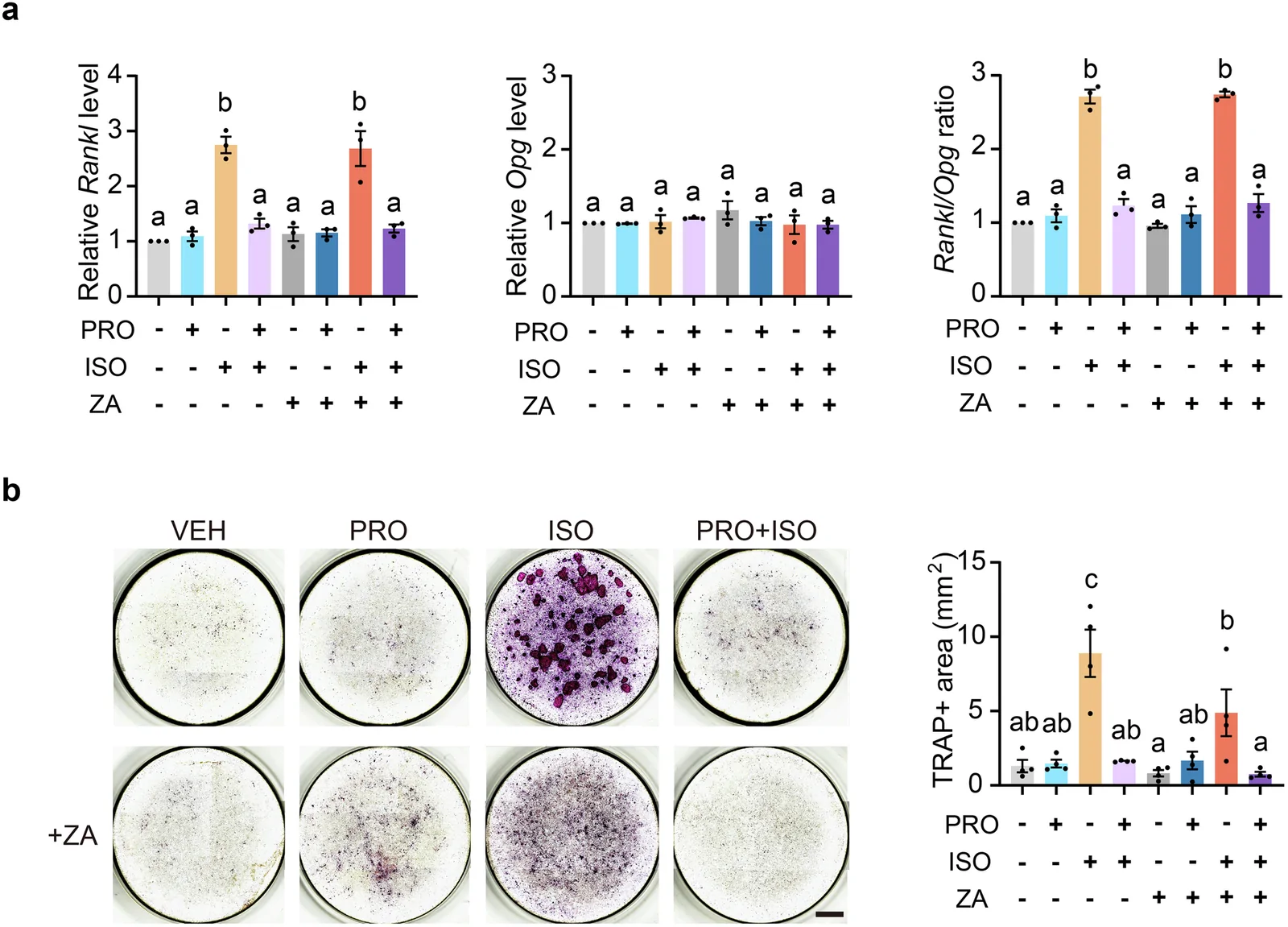
Propranolol additively suppresses osteoclastogenesis through a pathway distinct from that of zoledronic acid. **a** Primary osteoblasts were treated for 24 h with propranolol (PRO, 500 nM), isoproterenol (ISO, 100 nM) and/or zoledronic acid (ZA, 150 nM) as indicated. The relative mRNA expression levels of *Rankl* (*Tnfsf11*) and *Opg* (*Tnfrsf11b*) were quantified by quantitative PCR, and the *Rankl*/*Opg* ratio was calculated. Data are presented as fold changes relative to vehicle (VEH)-treated controls. **b** Representative images of tartrate-resistant acid phosphatase (TRAP) staining and quantitative analysis of the area of TRAP-positive multinucleated cells (mm^2^) in an osteoblast–osteoclast co-culture system treated with PRO, ISO and/or ZA. Scale bar, 10 μm. Quantitative data are shown as mean ± SEM. Different letters indicate statistically significant differences between groups (*P* < 0.05) as determined by one-way ANOVA followed by Tukey’s multiple comparison test.

Propranolol treatment alone did not significantly alter the *Rankl*/*Opg* ratio, indicating that basal β-adrenergic signalling is limited under these culture conditions (Fig. 5a). In contrast, isoproterenol significantly increased the *Rankl*/*Opg* ratio, and this effect was completely abolished by co-treatment with propranolol, confirming effective antagonism of β-adrenergic signalling. Zoledronic acid treatment did not affect the *Rankl*/*Opg* ratio, either alone or in combination with the adrenergic agents, suggesting that zoledronic acid does not directly modulate osteoblast-mediated osteoclastogenic signalling at the transcriptional level.

To functionally validate these findings, we finally examined osteoclast differentiation using an osteoblast–osteoclast co-culture system. Following sympathetic stimulation with isoproterenol, zoledronic acid markedly suppressed osteoclast differentiation, consistent with its direct antiresorptive effect (Fig. 5b). Importantly, the addition of propranolol further reduced osteoclast formation beyond the effect of zoledronic acid alone, suggesting an additive inhibitory effect. These results indicate that propranolol suppresses osteoclast differentiation through a mechanism distinct from that of zoledronate, and that combined β-adrenergic blockade and bisphosphonate treatment leads to excessive inhibition of osteoclastogenesis. This additive suppression may contribute to the aggravated MRONJ phenotype observed in propranolol-treated mice.

## Discussion

In this study, we identify circulating DBH as a novel candidate biomarker for MRONJ. Using serum proteomic profiling, we observed a significant reduction in circulating DBH levels in patients with MRONJ, which was validated in an independent clinical cohort and further supported by analyses in the UK Biobank population. In addition to its potential as a biomarker, our experimental findings suggest that impaired sympathetic signalling may exacerbate MRONJ pathology. Collectively, these findings support an association between reduced circulating DBH levels and MRONJ and provide evidence linking sympathetic dysregulation to MRONJ pathogenesis.

Several serum biomarkers, including CTx, TRACP5b, the RANKL/OPG ratio and DKK1, have been proposed for MRONJ; however, none has demonstrated sufficient clinical reliability. CTx, the earliest candidate biomarker^4^, was later shown by multiple meta-analyses to have limited predictive value and is no longer recommended in current guidelines^30,31^. Other reported markers, such as TRACP5b, DKK1 (ref.^6^), and the RANKL/OPG ratio^7^, were derived from small, single-centre and largely unreplicated studies, often using suboptimal control groups, which substantially limits their translational relevance. Consistent with these limitations, TRACP5b and DKK1 were not significant in our proteomic analysis (Supplementary Table 1). To address these limitations, we applied an unbiased serum proteomic approach in a rigorously defined cohort that included controls without MRONJ who were exposed to medication, followed by validation in an independent cohort and complementary analyses in the UK Biobank. This study design enabled the identification of circulating protein alterations that are reproducible across datasets and relevant to clinical MRONJ.

We demonstrated that reduced circulating DBH levels are associated with an increased risk of MRONJ. DBH is co-released with norepinephrine from sympathetic neurons and adrenal chromaffin cells^17,32,33^, but, unlike norepinephrine, it remains relatively stable in the circulation, making it a practical surrogate marker of sympathetic tone^16,34^. Although earlier physiological studies suggested that circulating DBH reflects sympathetic activation^17,35^, subsequent reports yielded inconsistent associations with blood pressure and other autonomic indices^18,19^, largely due to limited sample sizes and heterogeneous study designs. To clarify this issue, we examined plasma DBH levels in the large-scale UK Biobank cohort. After adjustment for major clinical and genetic confounders, DBH levels were significantly associated with systolic and diastolic blood pressure as well as hypertension status. These findings support the use of circulating DBH as an indicator of systemic sympathetic activity at the population level and suggest that reduced DBH levels in MRONJ patients may reflect altered sympathetic regulation.

Experimental and human genetic evidence further supports the physiological importance of DBH. Mice with targeted disruption of *Dbh* are unable to synthesize norepinephrine and epinephrine^36^, whereas patients with rare DBH deficiency exhibit markedly reduced or absent norepinephrine and epinephrine levels, elevated dopamine levels and severe autonomic dysfunction^37^. These studies collectively establish DBH as a key determinant of noradrenergic and sympathetic physiology. However, previous DBH genetic and deficiency studies have primarily focused on cardiovascular, autonomic and neuropsychiatric phenotypes, and have not directly examined MRONJ or jawbone pathology. Our findings extend this biological framework by linking reduced circulating DBH levels to MRONJ, a disease context in which sympathetic regulation has not been well explored.

To further explore the relationship between sympathetic tone and MRONJ, we pharmacologically inhibited sympathetic activity with propranolol in mice treated with zoledronic acid. It should be noted that propranolol administration does not represent a direct experimental model of reduced circulating DBH. Circulating DBH is interpreted as an indirect surrogate marker of sympathetic activity, whereas propranolol suppresses downstream β-adrenergic receptor signalling. Thus, the propranolol experiment was designed to functionally evaluate whether impaired β-adrenergic signalling, as suggested by reduced circulating DBH levels, could exacerbate MRONJ-associated bone pathology. Propranolol blocks β2-adrenergic signalling in bone, leading to reduced osteoblast-derived OPN and RANKL, suppression of osteoclast activity and concomitant promotion of osteoblast differentiation^38–41^. In our model, propranolol treatment markedly aggravated MRONJ lesions and was accompanied by a further reduction in TRAP-positive osteoclasts on top of zoledronic acid-induced suppression (Fig. 4h, i), indicating enhanced inhibition of bone resorption. Given that excessive suppression of bone resorption is widely regarded as a central mechanism in MRONJ pathogenesis^29^, these findings suggest that reduced sympathetic signalling may contribute to MRONJ progression, potentially through further suppression of osteoclast-mediated bone turnover.

Our findings differ from those of Du et al., who reported a protective effect of propranolol in MRONJ, a discrepancy that is most plausibly explained by differences in model design^42^. Notably, dexamethasone was included in their MRONJ induction protocol. As a potent glucocorticoid, dexamethasone exerts profound immunosuppressive and anti-inflammatory effects and is well known to impair bone healing, osteoclast function and stem-cell activity^43,44^. The inclusion of dexamethasone therefore represents a major confounding factor that complicates attribution of the observed effects specifically to propranolol^45^. In contrast, our zoledronic acid-only model more closely reflects a commonly used experimental framework for antiresorptive-associated MRONJ and enables evaluation of sympathetic blockade without additional immunomodulatory agents. Although Du et al. emphasized the potential bone-forming effects of propranolol^42^, accumulating evidence indicates that excessive suppression of bone resorption, rather than impaired bone formation, is the dominant driver of MRONJ pathogenesis^29^. This concept is further supported by clinical observations that romosozumab, despite its anabolic effects, slightly but significantly increases MRONJ risk^46,47^. Together, these observations suggest that under conditions of sympathetic blockade, enhanced suppression of bone resorption may contribute to MRONJ development.

Several limitations should be acknowledged. Although we addressed major methodological weaknesses of previous studies through well-characterized cohorts and independent validation, our sample size remains modest, and the clinical heterogeneity of MRONJ may limit the generalizability of our findings. Circulating DBH represents an indirect surrogate of sympathetic activity and may be influenced by DBH genetic polymorphisms and comorbid cardiovascular conditions. Accordingly, tissue-level validation of DBH and its integration with established autonomic markers, including norepinephrine, will be important to further enhance its clinical utility. Because DBH was measured after MRONJ onset, our study cannot determine whether reduced DBH levels precede disease development; therefore, prospective longitudinal studies are warranted to evaluate its predictive value before clinical manifestation. In addition, although widely used definitions in large population-based studies were applied and analyses were restricted to individuals with relevant medication exposure, the MRONJ case definitions in the UK Biobank have limited specificity and may have introduced a degree of classification bias; thus, these findings should be interpreted with caution. Despite these limitations, our findings provide convergent evidence from clinical cohorts, population-level analyses and experimental models supporting an association between reduced circulating DBH levels and MRONJ, and suggest a potential role of sympathetic dysregulation in its pathogenesis.

**Fig. 6:**
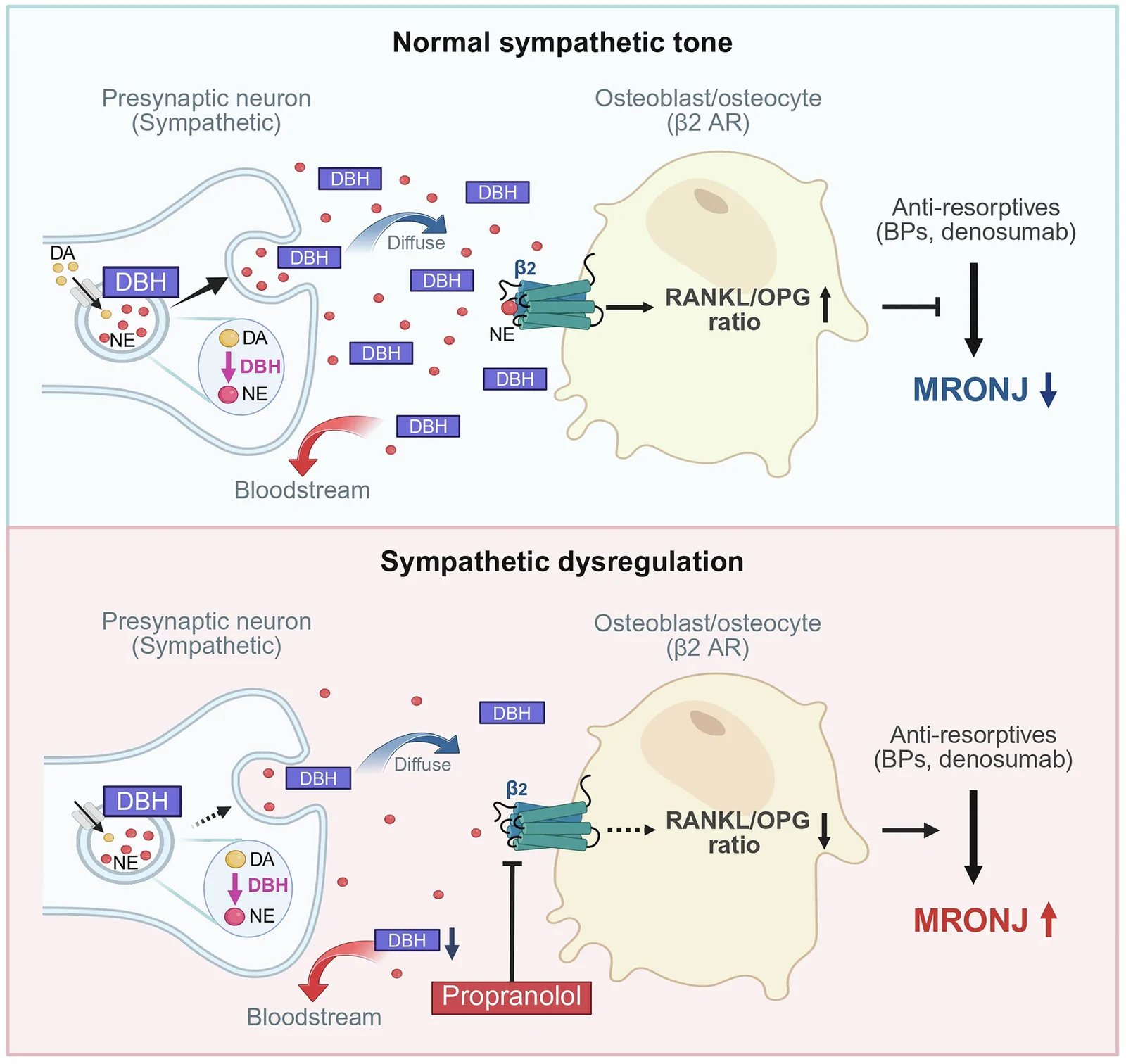
Proposed model of sympathetic dysregulation in the pathogenesis of medication-related osteonecrosis of the jaw. Under physiological conditions, dopamine β-hydroxylase (DBH) catalyses the conversion of dopamine to norepinephrine in presynaptic sympathetic neurons, maintaining sympathetic tone and supporting osteoblast-mediated osteoclastogenic signalling through balanced RANKL/OPG expression. DBH can diffuse into the synaptic cleft and partially enter the bloodstream, where circulating DBH reflects systemic sympathetic activity. In MRONJ, reduced circulating DBH indicates sympathetic downregulation, leading to decreased RANKL/OPG signalling and excessive suppression of osteoclast differentiation. Pharmacological β-adrenergic blockade with propranolol further amplifies this effect, resulting in additive inhibition of osteoclast activity in combination with bisphosphonates (BPs) and contributing to jawbone necrosis. This figure was created using BioRender.com. DA, dopamine; NE, norepinephrine; β2 AR, β2-adrenergic receptor.

Based on these integrated findings, we propose a conceptual framework in which circulating DBH may serve as a biomarker of sympathetic regulation in MRONJ pathogenesis (Fig. 6). Reduced DBH levels may reflect altered sympathetic activity, which could influence osteoblast-mediated RANKL/OPG signalling, promote excessive suppression of osteoclast activity and increase the vulnerability of the jawbone to necrosis. Pharmacological β-adrenergic blockade further perturbed this balance in our experimental model, providing a potential mechanistic explanation for the aggravated MRONJ phenotype observed in propranolol-treated mice. Taken together, our findings support an association between reduced circulating DBH levels and MRONJ and suggest that sympathetic dysregulation may contribute to disease progression. By linking sympathetic signalling to MRONJ-related bone pathology, this study provides new insights into the neuro-skeletal regulation of jawbone homeostasis and highlights circulating DBH as a candidate biomarker for further evaluation in risk assessment and disease monitoring in patients receiving antiresorptive therapy.

## Supplementary information

Supplementary Information

Supplementary Table 1
