# Supplementary material for: Decreased circulating dopamine β-hydroxylase is associated with medication-related osteonecrosis of the jaw and implicates sympathetic dysregulation
Source: Exp Mol Med. 2026 Oct 14;58(10):3565–78. doi: 10.1038/s12276-026-01868-z (PMC13737051; doi:10.1038/s12276-026-01868-z)
Supplement: Supplementary file 1 [file 12276_2026_1868_MOESM1_ESM.pdf]

Supplementary Fig. 1. Sensitivity analyses of circulating DBH levels and MRONJ in clinical cohorts

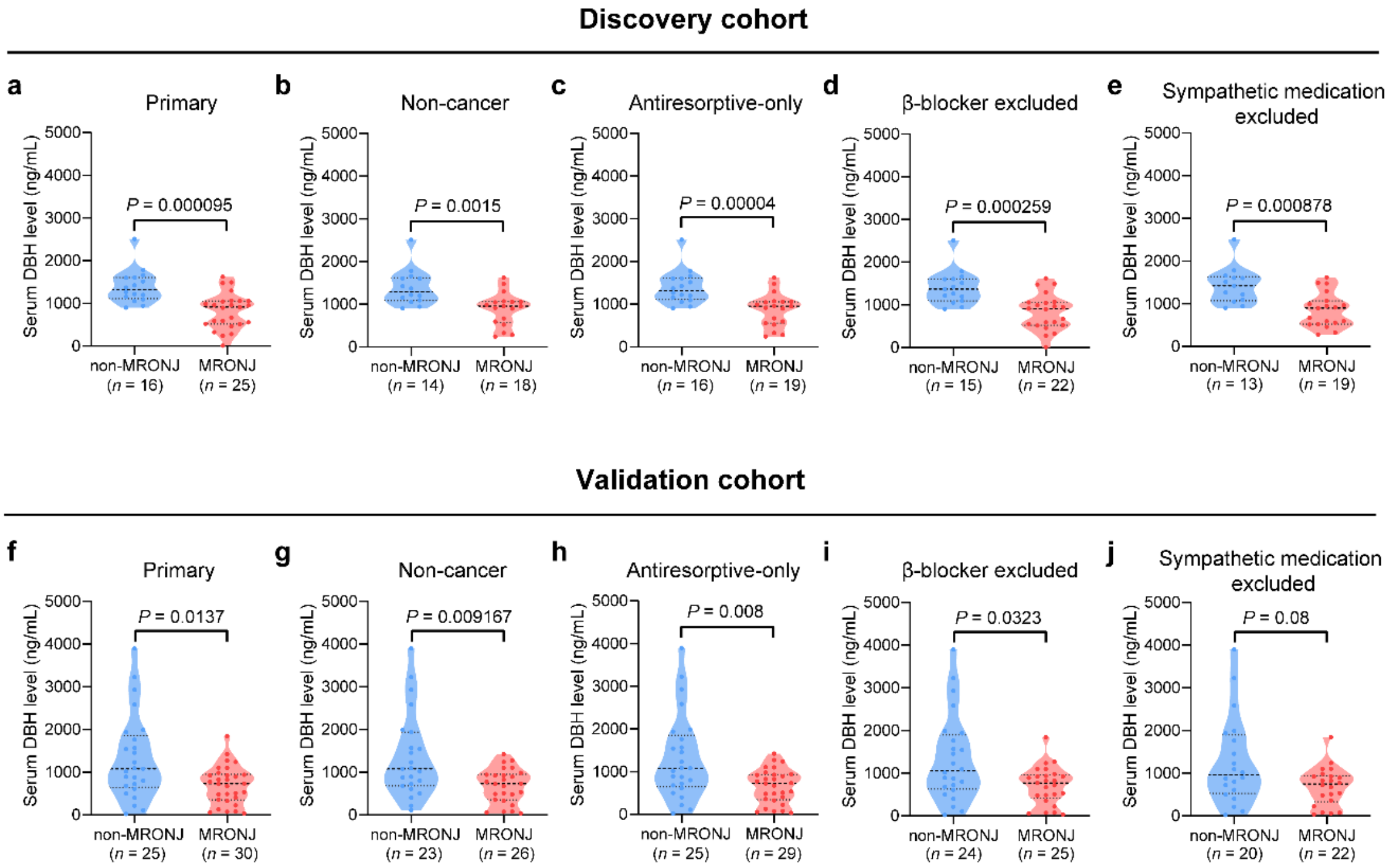

(a–e) Discovery cohort and (f–j) independent validation cohort. (a,f) Primary analysis. (b,g) Patients without cancer. (c,h) Patients receiving antiresorptive therapy without concomitant antiangiogenic agents. (d,i) Analysis after excluding β-blocker users. (e,j) Analysis after excluding users of any sympathetic nervous system–affecting medications. Violin plots represent the distribution, with center lines indicating the median and interquartile range. Statistical significance was assessed using a two-sided Mann–Whitney test.

Supplementary Fig. 2. Participant selection for MRONJ analysis in the UK Biobank plasma proteomics cohort.

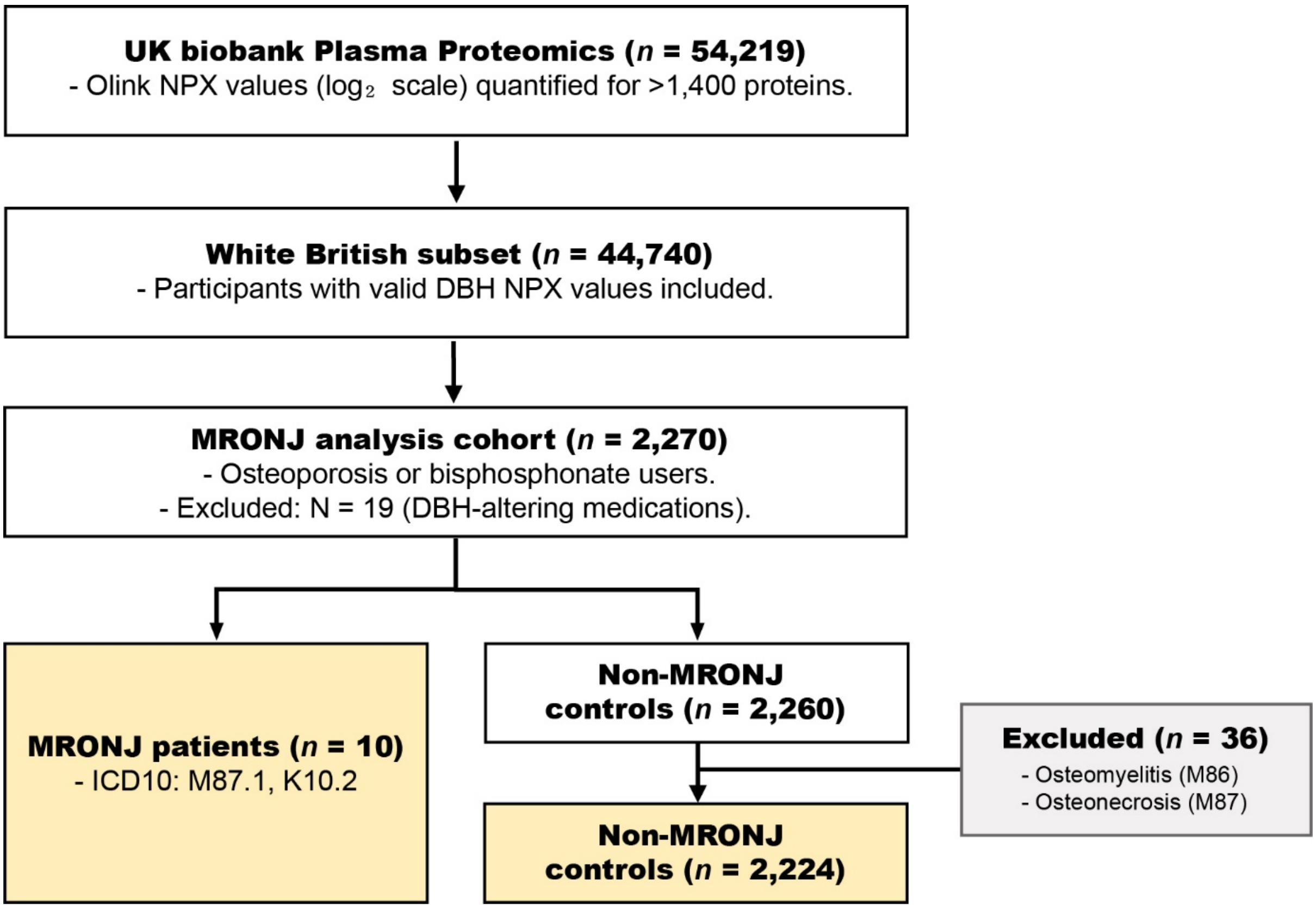

Flow diagram showing the inclusion and exclusion criteria used to define MRONJ patients and non-MRONJ controls from the UK Biobank plasma proteomics dataset.

Supplementary Fig. 3. Propranolol aggravates systemic bone remodeling alterations in zoledronic acid–treated mice.

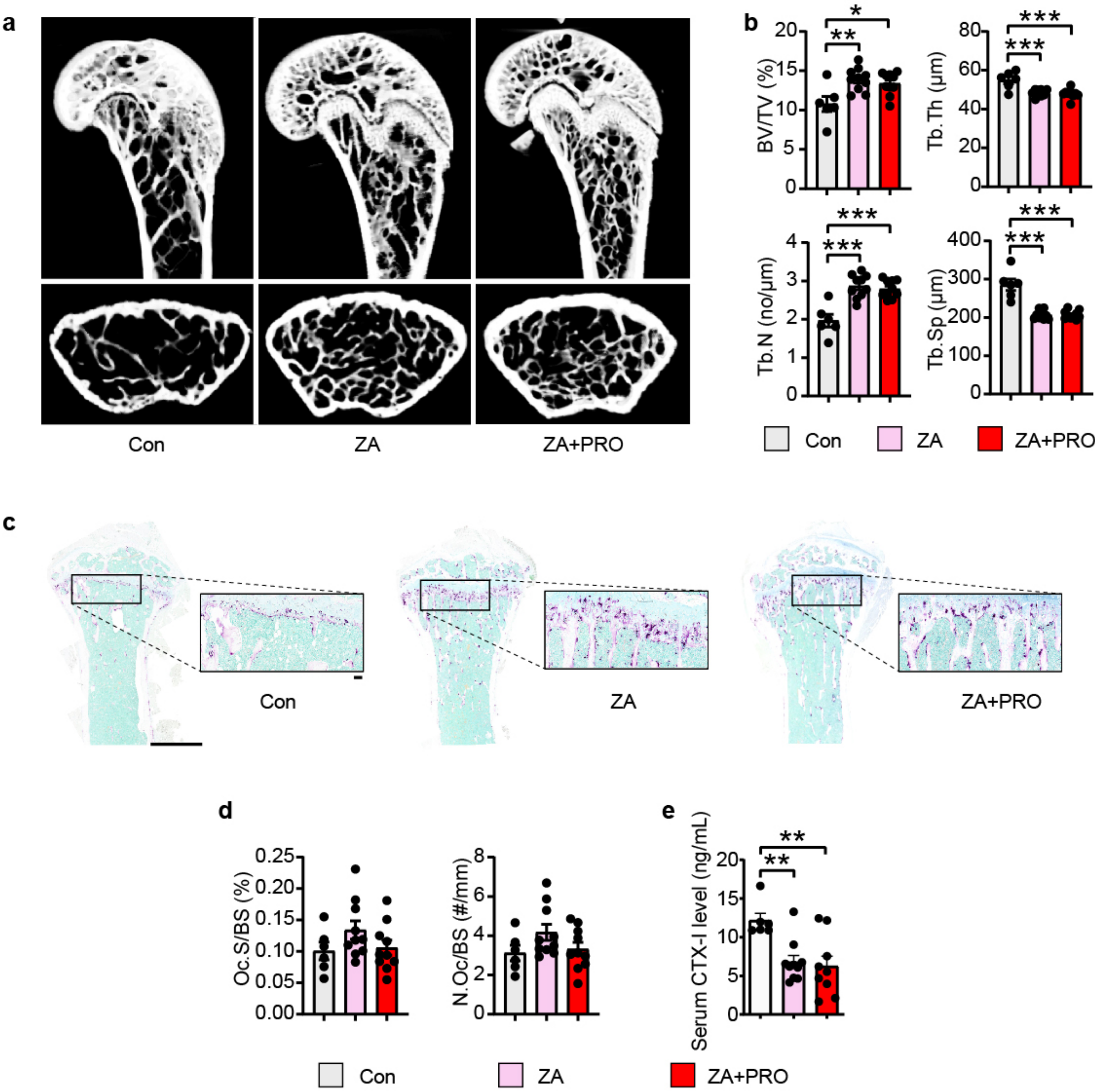

a Representative micro-computed tomography (micro-CT) images of the femur in control (Con), zoledronic acid–treated (ZA), and ZA plus propranolol–treated (ZA+PRO) mice.

b Quantitative micro-CT analysis of femoral trabecular bone parameters, including bone volume fraction (BV/TV), trabecular thickness (Tb.Th), trabecular number (Tb.N), and trabecular separation (Tb.Sp).

c Representative tartrate-resistant acid phosphatase (TRAP)-stained sections of the tibia in Con, ZA, and ZA+PRO groups.

d Quantification of osteoclast surface per bone surface (Oc.S/BS) and osteoclast number per bone surface (N.Oc/BS) in tibial sections.

e Serum levels of C-terminal telopeptide of type I collagen (CTX-I) measured by ELISA.

Data are presented as mean ± SEM. $^{*}P < 0.05$, $^{**}P < 0.01$, $^{***}P < 0.001$.

Supplementary Table 2. Multivariable logistic regression analyses of circulating DBH levels and MRONJ in sensitivity subsets across discovery and independent validation cohorts

Cohort	Subset	MRONJ/control, *n*	OR[‡]	95% CI	*P*-value
Discovery	Primary	25 / 16	11.68	1.99-68.37	0.006
	Non-cancer	18 / 14	7.67	1.45-40.58	0.017
	Antiresorptive-only	19 / 16	9.80	1.80-53.23	0.008
	Excluding β-blocker users	22 / 15	9.98	1.80-55.48	0.009
	Excluding sympathetic nervous system–affecting medication users	19 / 13	8.51	1.62-44.84	0.012
Validation	Primary	30 / 25	2.52	1.09-5.81	0.031
	Non-cancer	26 / 23	3.29	1.09-9.93	0.035
	Antiresorptive-only	29 / 25	3.18	1.18-8.55	0.022
	Excluding β-blocker users	25 / 24	2.37	0.98-5.71	0.055
	Excluding sympathetic nervous system–affecting medication users	22 / 20	2.19	0.87-5.51	0.094

Odds ratios (ORs) and 95% confidence intervals (CIs) were estimated using logistic regression models adjusted for age and sex. The number of MRONJ patients and non-MRONJ controls included in each subset is shown as MRONJ/control, *n*. DBH levels were standardized and modeled per 1 standard deviation (SD) decrease. Sympathetic nervous system–affecting medication users were defined as participants taking β-blockers, α-blockers, central sympatholytics, sympathomimetic agents, serotonin–norepinephrine reuptake inhibitors, tricyclic antidepressants, monoamine oxidase inhibitors, or dopaminergic medications.

Supplementary Table 3. Clinical characteristics of MRONJ patients and non-MRONJ controls in the UK Biobank

Characteristic	MRONJ patients (*n* = 10)	non-MRONJ controls (*n* = 2,224)	*P*-value‡
**Demographics**			
Age, yrs	59.9 ± 9.24	61.70 ± 6.09	0.554
Female	7 (70)	1806 (81.2)	0.411
**Clinical history**			
Osteoporosis	9 (90)	1850 (83.2)	1
ONJ-related cancer†	0 (0)	1 (0.04)	1
Hypertension	5 (50)	1337 (60.1)	0.533
Diabetes	1 (10)	101 (4.5)	0.374
**Medication exposure**			
Alendronate	2 (20)	538 (24.2)	1
Ibandronate	1 (10)	31 (1.4)	0.135
Zoledronic acid	0 (0)	6 (0.3)	1
Risedronate	0 (0)	128 (5.8)	1
Etidronate	0 (0)	18 (0.8)	1
Clodronate	0 (0)	8 (0.4)	1
Palmidronate	0 (0)	3 (0.1)	1
Overall BP	3 (30)	730 (32.8)	1

Values are mean ± SD or number (%).† Defined as prostate cancer, breast cancer, multiple myeloma, or lung cancer treated with zoledronic acid. Age compared with Welch's t-test; all categorical variables with Fisher's exact test (two-sided).

Supplementary Table 4. Oligonucleotide primers

Target	Forward primer sequence (5'→3')	Reverse primer sequence (5'→3')	Gene bank accession
Tnfsf11 (RANKL)	ATC CCA TCG GGT TCC CAT AA	TCC GTT GCT TAA CGT CAT GTT AG	NM_011613.4
Tnfrsf11b (OPG)	TAC CTG GAG ATC GAA TTC TGC TT	CCA TCT GGA CAT TTT TT GCA AA	NM_008764.4
Gapdh	AGG TCG GTG TGA ACG GAT TTG	TGT AGA CCA TGT AGT TGA GGT CA	NM_001411840.1

All primers were designed based on mouse mRNA sequences obtained from the NCBI Gene database, and their specificity was verified by BLAST analysis. Gene expression levels were normalized to *Gapdh* as an internal control.
